# From Pseudocyclic to Macrocyclic Ionophores: Strategies toward the Synthesis of Cyclic Monensin Derivatives

**DOI:** 10.1021/acs.joc.4c02715

**Published:** 2025-01-10

**Authors:** Michał Sulik, Robert Graniczny, Jan Janczak, Dagmara Kłopotowska, Joanna Wietrzyk, Adam Huczyński

**Affiliations:** † Department of Medical Chemistry, Faculty of Chemistry, Adam Mickiewicz University, Uniwersytetu Poznańskiego 8, 61−614 Poznań, Poland; ‡ Institute of Low Temperature and Structure Research, Polish Academy of Sciences, Okólna 2, Wrocław 50−422, Poland; § Hirszfeld Institute of Immunology and Experimental Therapy, Polish Academy of Sciences, Rudolfa Weigla 12, 53−114 Wrocław, Poland

## Abstract

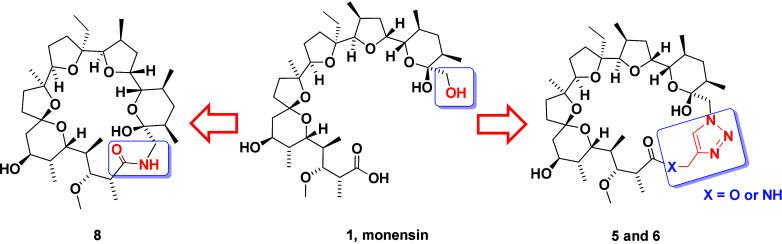

There has been a long search for a simple preparation of new cyclic analogues of ionophore antibiotics. We report a simple and general synthesis of three new cyclic derivatives of polyether ionophore, monensin A (MON). The application of the Huisgen 1,3-dipolar cycloaddition of azides and terminal alkynes to macrocyclization results in a concise, synthetic route to monensin lacton or lactam in only 4 steps. Additionally, macrolactamization by a simple amidation reaction using HATU, a commonly used conjugating agent, gives 72% yields and utilizes neither high dilution techniques nor template effects in the cyclization step. This in turn enables ready access to a range of unnatural MON analogues, showing the ability to form complexes with monovalent and divalent metal cations.

## Introduction

**Figure 1:**
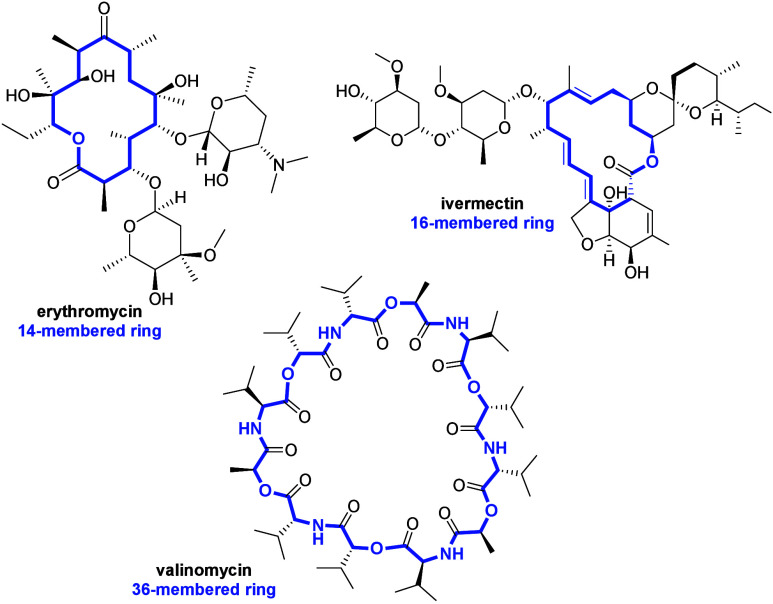
Chemical structures of erythromycin, ivermectin, and valinomycin.

Among the numerous drugs used in modern medicine, many of them belong to the broad group of macrocyclic compounds.^1,2^ There are many examples of natural macrocycles, including erythromycin–a macrolide antibiotic used for the treatment of a number of bacterial infections, ivermectin–a macrocyclic lactone used in the treatment of many parasitic diseases, and valinomycin–dodecadepsipeptide being a representative of natural, cyclic ionophores (Figure 1).^3−7^

In 1967, Pressman et al. proved that valinomycin was capable of coordinating potassium cations and transporting them across biological membranes.^8^ This discovery is considered to be the beginning of chemistry of ionophores which has been a very intensively studied group of compounds ever since. To date, hundreds of ionophores have been described.^9,10^ Their ability to complex metal cations makes them useful in analytical chemistry as components of ion-selective electrodes, and in organic synthesis as a phase transfer catalysts.^11,12^

**Figure 2:**
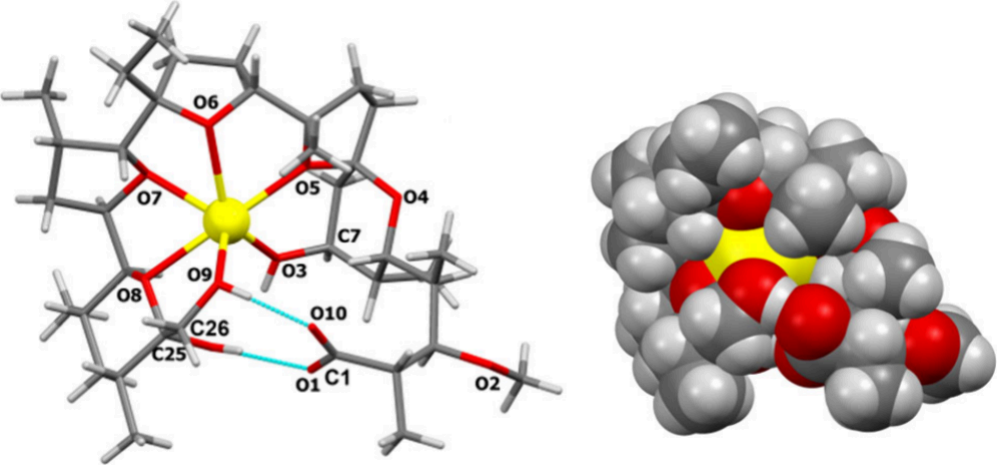
Wireframe representation and space-filling presentation of the crystal structure of monensin A sodium salt (This figure was reproduced using data downloaded from the Cambridge Crystallographic Data Centre CCDC: 620458).

Moreover, due to the ability to transport metal cations across biological membranes, these compounds exhibit a wide spectrum of biological activities, including antibacterial, antiparasitic, and anticancer ones.^13,14^ Among the ionophores that are currently of greatest interest in medicinal chemistry, attention should be paid to the group of noncyclic carboxylic ionophores. These compounds, whose main representative is monensin A (**MON**), demonstrate the ability to imitate cyclic ionophores due to the presence of intramolecular hydrogen bonds in their structure (Figure 2).^15,16^

The pseudocyclic structure facilitates **MON** coordination of metal cations (with the highest selectivity toward the sodium cation) and transport them across cell membranes, which leads to the disturbance in the intracellular pH and ion concentration gradient, and subsequently to mitochondrial damage, vacuolization, and cell death (Figure 2).^15,16^

To date, many **MON** derivatives have been synthesized by the chemical modification of its C1 carboxyl group or the hydroxyl group attached to the C26 carbon atom.^17−23^ However, an interesting and still undeveloped research direction is the cyclization of the **MON** molecule, which should allow the transformation of its pseudocyclic skeleton into a macrocyclic structure, leading to a change in its structural properties, including the number of rotatable bonds and polar surface area.^6^ Derivatives of this type have already been obtained applying the Corey–Nicolaou macrolactonization reaction using 2,2′-dipyridyl disulfide and triphenylphosphine on unmodified **MON** or its amides with chiral amino acids.^15,21,24−26^ It is worth noting that historically **MON** is one of the first natural compounds ever to be macrocyclized.

Noteworthy, all macrocyclic **MON** derivatives synthesized so far belong to the group of lactones. It should be noted that under physiological conditions, esters can undergo hydrolysis due to the presence of esterases.^27^ Therefore, we decided to synthesize more stable macrocyclic **MON** derivatives by applying an amide bond and/or a 1,2,3-triazole ring.

## Results and Discussion

**Scheme 1:**
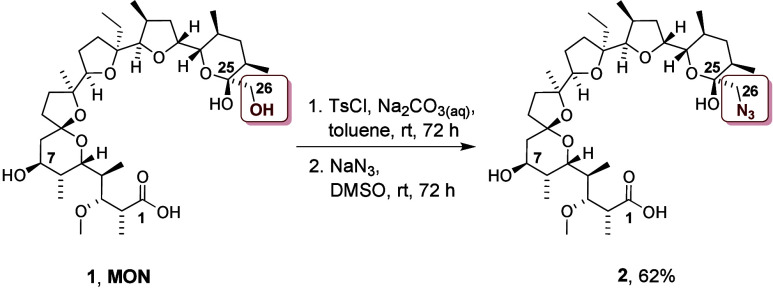
Synthesis of MON Azide (Compound **2**) Reaction conditions and yield are indicated

To the best of our knowledge, despite many modifications performed on the C26 hydroxyl group of **MON**, its conversion into an azide group has not been established yet. After conducting a variety of chemical reactions, we present the first scalable, efficient, and simple method for obtaining C26 azide of **MON** (Scheme 1). The first step involves the tosylation of the C26-hydroxyl group of **MON**, and then the resulting tosylate is directly subjected to the nucleophilic substitution reaction with the excess of sodium azide (Scheme 1). The obtained C26-azide of **MON** (compound **2**) was purified chromatographically. Having access to the azide functionality (compound **2**), an application of Nobel prize-honored distinction Meldal and Sharpless click reaction in the synthesis of new macrocyclic **MON** derivatives was a natural step. The 1,2,3-triazole linker is a very attractive bioisostere in medicinal chemistry, which both structurally and electronically mimics the amide bond.^28,29^

**Scheme 2:**
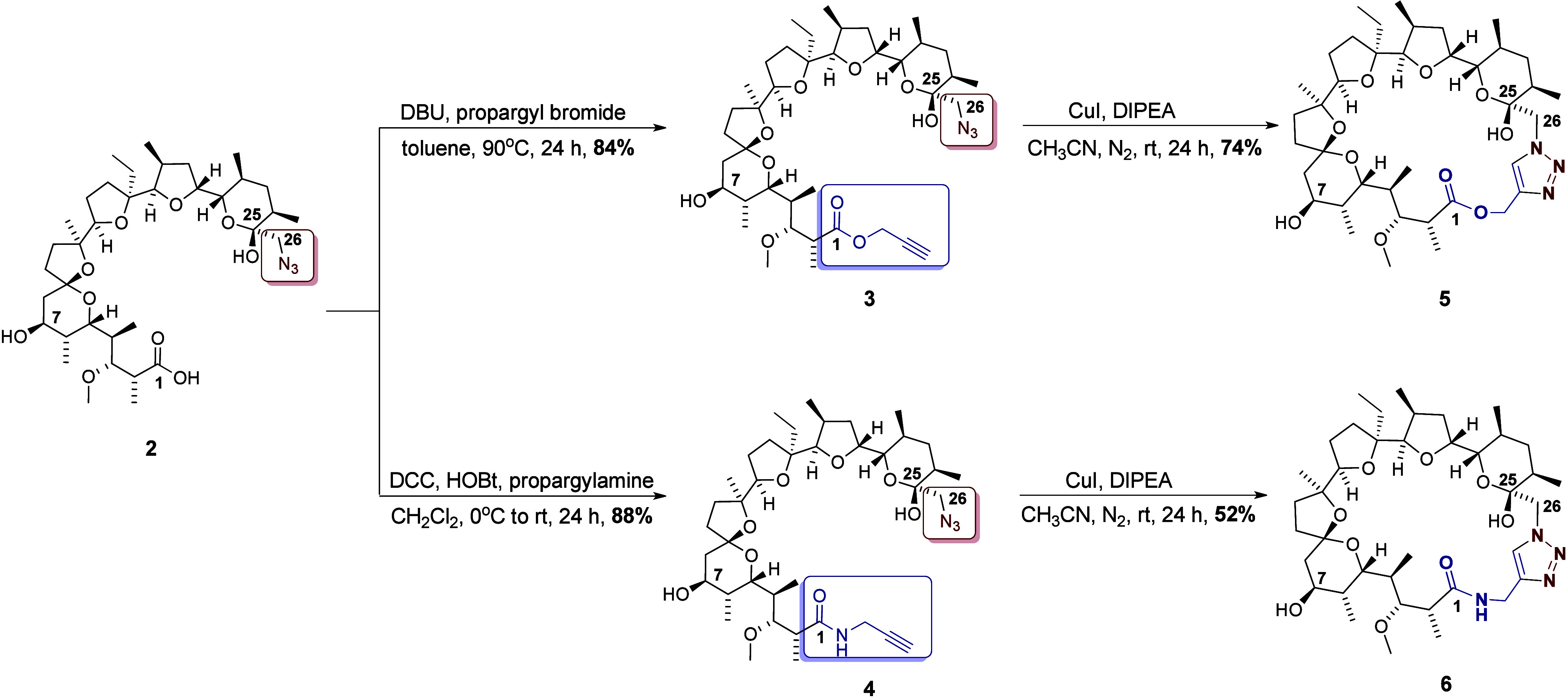
Synthesis of MON Macrocycles Using the Click Reaction Reaction conditions and yields are indicated

In the context of chemical modification of ionophores by click chemistry, the most suitable and efficient method is the application of Meldal CuAAC reaction, which involves the use of copper(I) iodide.^17,30^ To conduct the intramolecular click reaction, which will enable the formation of the macrocyclic system, it was necessary to introduce a triple bond into the structure of compound **2**. We decided to synthesize both, the propargyl amide and ester of **MON** azide (Scheme 2). Enzymatic stability may have a crucial effect on the biological, and structural properties of the obtained macrocycles, thus this distinction will allow us to conclude whether there is a correlation between the structure, and the activity of obtained analogues (structure–activity relationship, SAR).

The obtained precursors (ester **3** and amide **4**) were dissolved in acetonitrile, then DIPEA and copper(I) iodide were added. The reactions were carried out in an inert gas atmosphere to avoid oxidation of copper(I) iodide. Both click reactions had to be performed using a large amount of solvent (high dilution) to reduce the risk of an intermolecular Meldal reaction leading to polymerization. When the reaction was conducted with a high substrate concentration, a precipitate was formed. It was insoluble in most organic and inorganic solvents. The use of high dilution conditions allowed us to eliminate the formation of this polymeric byproduct. Both products were purified chromatographically, with a yield of 52% (for **6**) and 74% (for **5**), respectively (Scheme 2).

**Scheme 3:**
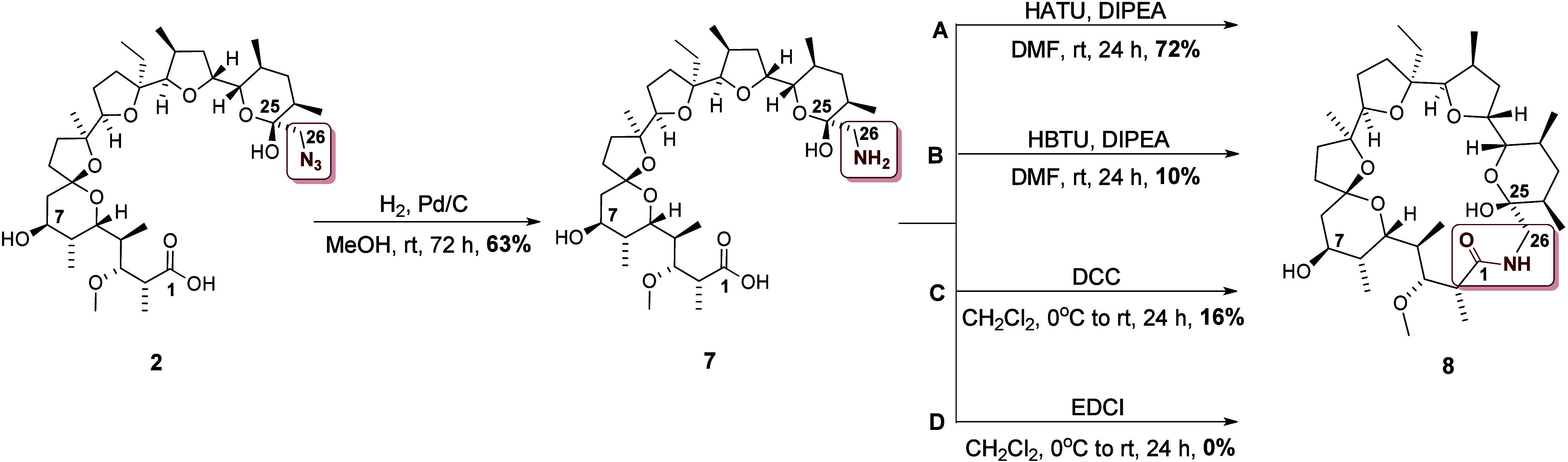
Synthesis of MON Amine (Compound **7**) and Its Macrocyclic Lactam Derivative (Compound **8**) Reaction conditions and yields are indicated

In the next step we decided to perform macrocyclization by introducing an amide bond. However, the method of synthesis of the C26 amine of **MON** has not been described so far. In order to obtain the amine, we decided to carry out the reduction of the azide group of **2**. It is worth noting that this compound does not have multiple bonds in its structure, nor other functional groups sensitive to reduction in the presence of hydrogen, so this reductive reagent can be successfully used (Scheme 3). The resulting amine (compound **7**) was purified chromatographically and used as a substrate in the macrocyclization reaction.

Compound **7**, which has both a carboxyl and an amino group in its structure, can be treated as an amino acid. Therefore, the reagents used for peptide synthesis can be applied for the synthesis of the macrocyclic lactam of **MON**. For this purpose we selected the four most commonly used conjugating agents: HATU, HBTU, DCC, and EDCI (Scheme 3). As can be noted, the most effective method of macrolactamization was the use of HATU on the starting amino acid **7** (Scheme 3). The yield of this reaction (72%) was several times higher than in the case of other reagents used. Interestingly, the formation of the desired product **8** was not observed in the reaction with EDCI (Scheme 3).

The structure and purity of the obtained **MON** derivatives were determined by spectroscopic (NMR, FT-IR) and spectrometric (ESI-HRMS and ESI-MS) methods. In addition, compounds **2**, **5**, and **8** were obtained in crystalline form, which enabled scXRD analysis. In the ^13^C NMR spectra of **MON** macrocycles, the signals of the highest analytical significance were assigned to the carbonyl group of the ester/amide moiety at position C1. These signals appeared in the range of 175.5–178.3 ppm. Moreover, for compounds **5** and **6** we observed the appearance of a signal from the quaternary carbon atom of the triazole ring, in the range of 142.9–144.9 ppm. In the ^1^H NMR spectra of lactams **6** and **8**, we observed a diffuse signal from the amide group in the range of 6.50–7.41 ppm. In addition, for click derivatives (compounds **5** and **6**), a characteristic, intensive singlet appeared, originating from the hydrogen atom of the triazole ring in the range of 7.84–7.90 ppm. Furthermore, for compounds of pseudocyclic nature (compounds **2**, **3**, **4**, and **7**), the diagnostic signals are two characteristic doublets originating from the methylene group at the C26 position. We observed them in the range of 3.05–3.08 ppm, and 3.15–3.48 ppm, respectively. In the FT-IR spectra, the most distinctive signals originate from the stretching vibrations of the azide group (νN_3_), which for the azide derivatives **3** and **4** appeared at 2102 cm^–1^. Importantly, for the click reaction products, the signals at this value disappeared, which confirms that the reaction occurred in the expected direction. Moreover, for esters (compounds **3**, and **5**) the characteristic signal from the stretching vibrations of the ester carbonyl group appears in the narrow range of 1741–1744 cm^–1^. The signal originating from the stretching vibrations of the amide carbonyl group (compounds **4**, **6**, and **8**) appears in the range of 1641–1657 cm^–1^. The detailed synthesis procedures, as well as NMR, ESI-MS, and FT-IR spectra of the obtained macrocyclic derivatives, are included in the Supporting Information (Figures S1–S31).

**Figure 3:**
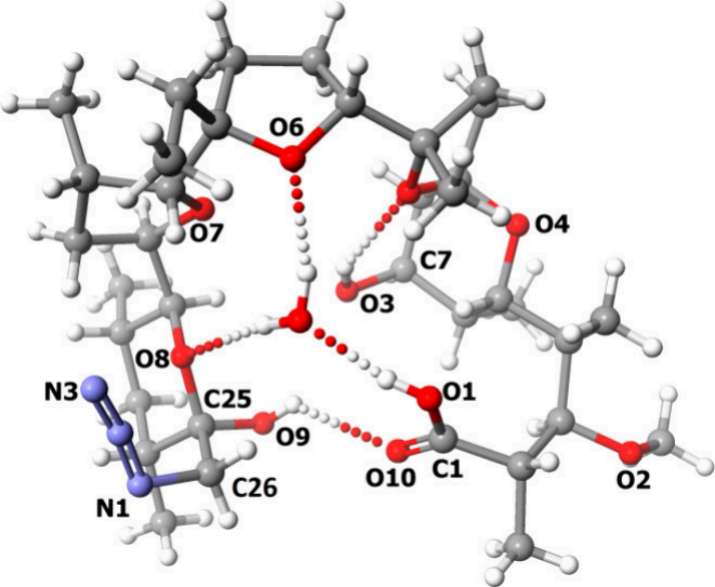
Crystal structure of the azide **2**–water complex.

Confirmation of the structure of the obtained derivatives and explanation of their structural properties is possible on the basis of the X-ray diffraction analysis. Thus, three of the obtained derivatives–**MON** azide (compound **2**), macrocyclic lactone (compound **5**), and macrocyclic lactam (compound **8**) were characterized using the X-ray single crystal diffraction method. Therefore, we were able to compare the structural properties of the pseudocyclic **MON** derivative with those of macrocyclic derivatives. Detailed crystal data, refinement parameters and experimental details for all molecules are presented in Table S1 and Figures S32–S37 (Supporting Information). Single crystals of compound **2** were grown by crystallization in acetonitrile solution. The azide crystallized in the noncentrosymmetric space group *P*2_1_ of the monoclinic system, as a solvate with the water molecule. The asymmetric unit of the compound **2**–water complex is illustrated in Figure 3.

**Figure 4:**
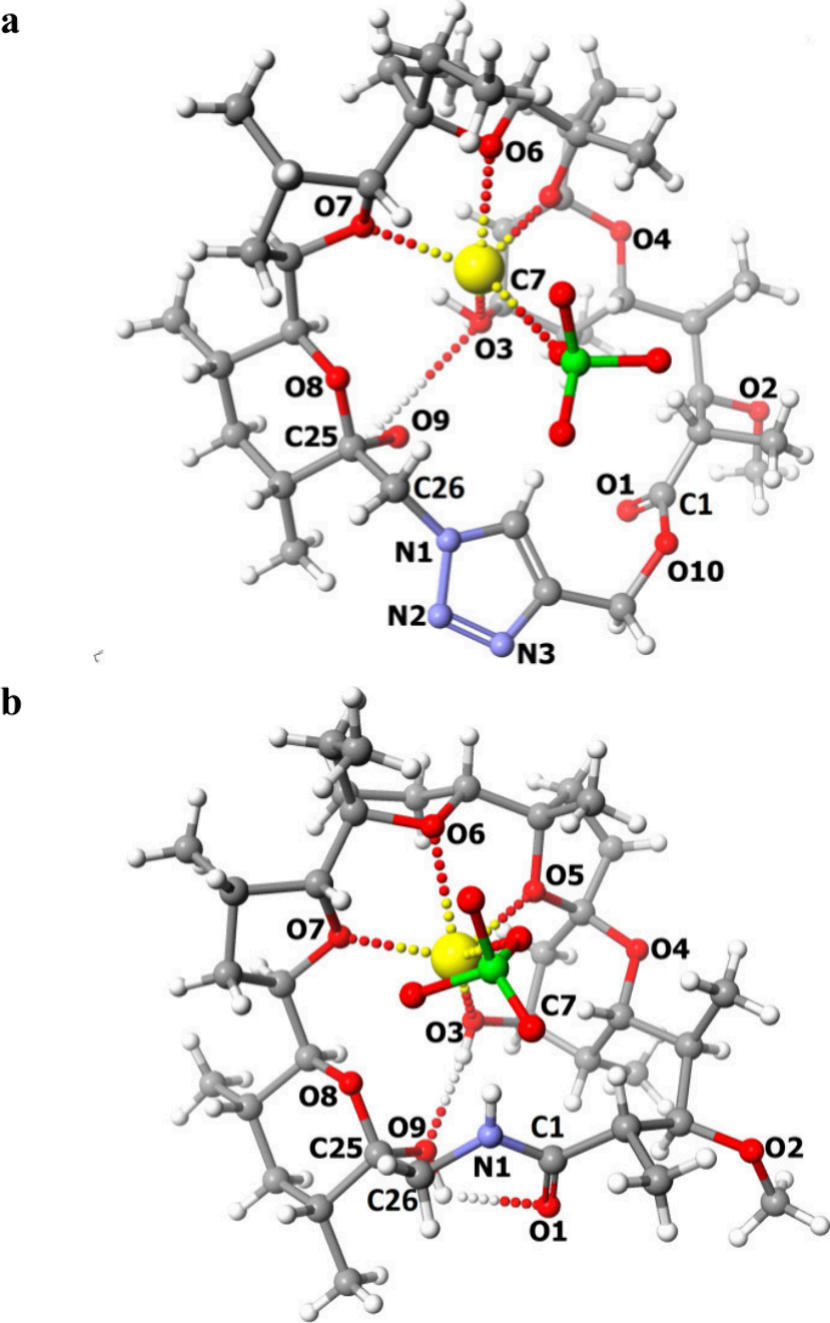
Crystal structure of: (**a**) sodium perchlorate–monensin lactone **5** complex, (**b**) sodium perchlorate–monensin lactam **8** complex.

The molecule of compound **2** in the crystal exhibits pseudocyclic conformation, with the azide substituent directed outside the polar pocket of the molecule. The structure is stabilized by several hydrogen bonds, whose exact geometry is described in the Supporting Information (Table S2). An example is the hydrogen bond O9—H9*A*···O10 formed between a carboxyl group and a hydroxyl group attached to the C25 carbon atom (Figure 3). Noteworthy, the water molecule stabilizes the pseudocyclic structure of compound **2** by forming additional hydrogen bonds with the oxygen atoms O6 and O8 (as donor) and the oxygen atom O1 (as acceptor) (Figure 3). The atom numbering and additional structural aspects are presented in the Supporting Information Figure S32. Single crystals of compound **5** were grown by crystallization in acetonitrile solution with the addition of NaClO_4_ (1.25 equiv. according to compound **5**). The lactone crystallized in the noncentrosymmetric space group *P*2_1_ of the monoclinic system, as a complex with a sodium cation and a perchlorate as a counterion, and as a solvate with the acetonitrile molecule. The asymmetric unit of the compound **5**–sodium perchlorate complex is illustrated in Figure 4a.

The scXRD analysis confirmed the cyclic structure of the compound. It is worth noting that the nitrogen atoms of the triazole ring are directed outside the polar pocket of the molecule and do not participate in coordinating the sodium cation, which may be a natural consequence of the conformation of the azido group in compound **2**. The structure is stabilized by a few hydrogen bonds, mainly O3—H3···O9 formed between the hydroxyl group attached to C25 carbon atom and the hydroxyl group attached to C7 carbon atom (Figure 4a). The hydroxyl group O9—H9 additionally forms hydrogen bonds with the perchlorate ion. The exact geometry of hydrogen bonds is described in the Supporting Information (Table S3). In addition, the perchlorate ion complements the coordination space of the sodium cation, thus stabilizing the resulting complex.

A significant change in structural properties is observed in compound **8**, which is a macrocyclic lactam of **MON**. Single crystals of compound **8** were grown by crystallization in acetonitrile solution with the addition of NaClO_4_ (1.25 equiv. according to compound **8**). The lactam crystallized in the noncentrosymmetric space group *P*2_1_2_1_2_1_ of the orthorhombic system, as a complex with a sodium cation and a perchlorate as a counterion. The asymmetric unit of compound **8**–sodium perchlorate complex is illustrated in Figure 4b.

The molecule of compound **8** belongs to the group of macrocyclic compounds. The structure is additionally stabilized by hydrogen bonds, however, their number is much smaller than in the case of the pseudocyclic derivative **2**. The exact geometry of hydrogen bonds is described in the Supporting Information (Table S4). Except for the O3—H3···O9 hydrogen bond formed between the hydroxyl group attached to C25 carbon atom and the hydroxyl group attached to C7 carbon atom, all the other hydrogen bonds are formed by the amide group being the donor. Interestingly, no hydrogen bonds are formed with the perchlorate ion, acting as the counterion, however, it stabilizes the structure by complementing the coordination space of the sodium cation.

In the FT–IR spectra of compound **8**, we observe a significant decrease in the wavenumber value for the stretching vibrations originating from the amide group after coordination with NaClO_4_ (1650 cm^–1^ for **8** versus 1641 cm^–1^ for **8** with NaClO_4_). Since the amide group is not involved in coordinating the sodium cation, it should be concluded that the presence of the molecule in the form of a complex additionally facilitates the formation of numerous hydrogen bonds by the amide group, which leads to the decrease in the wavenumber (Figures S30–S31). The atom numbering and additional structural aspects for compounds **5** and **8** are presented in the Supporting Information Figures S33–S34.

In addition to examining the structural properties of the obtained compounds, their ability to form complexes with metal cations is also important. Thanks to this property, ionophores can be widely used in analytical chemistry and organic catalysis, but also in medicinal chemistry, as compounds displaying potent biological activity. As mentioned above, ionophores form complexes with metal cations and transport them across the cellular lipid membranes, thus killing the cells by dissipating their membrane potential. **MON** exhibits a high affinity to the sodium cation, but this does not necessarily apply to its macrocyclic derivatives. The coordination space of these compounds is different from that of **MON**, which may cause a significant change in selectivity in coordinating metal cations. For this purpose, coordination assays were performed for **MON** macrocyclic derivatives **5**, **6**, and **8**.

We studied the ability to form complexes of cyclic **MON** derivatives with mono- (Li^+^, Na^+^, K^+^, Rb^+^, and Cs^+^) and divalent (Mg^2+^, Ca^2+^, Sr^2+^, and Ba^2+^) cations using electrospray ionization mass spectrometry (ESI-MS) because it permitted analysis of host–guest complexes and other noncovalent complexes formed in solution (Supporting Information, Figures S15–S23).^31^ The ESI-MS spectra prove the formation of the respective [**5** + M]^+^, [**5** + M + H_2_O]^+^, [**6** + M]^+^, [**8** + M]^+^, complexes, where M = Li, Na, K, Rb, and Cs.

On the other hand, the signals in the ESI-MS spectra of the complexes formed between **5**, **6**, or **8** and appropriate divalent metal perchlorates M(ClO_4_)_2_, where M = Mg, Ca, Sr and Ba, demonstrate the presence of the [**5** + MClO_4_]^+^, [**5** + MClO_4_ + H_2_O]^+^, [**6** + MClO_4_]^+^, [**8** + MClO_4_]^+^, and [**8**–H + MClO_4_]^+^ ion pair complexes probably formed due to the ion pairing between divalent metal–cation and perchlorate anion that occurs in acetonitrile solution and gas phase. Moreover, the interaction of Na^+^ cation with perchlorate anion has also been observed in the crystalline complexes of **5** and **8** (Figure 5). Therefore, scXRD and ESI-MS studies show that cyclic derivatives of **MON** should be recognized as ion pair receptors.^32^

Furthermore, to assess how macrocyclization affects the biological activity of **MON** analogues, we evaluated the antiproliferative activity of the obtained derivatives. Various cell lines were selected for the study, including human lung carcinoma (A549), human breast adenocarcinoma (MCF-7), human colon adenocarcinoma (LoVo), its doxorubicin-resistant subline (LoVo/DX), and normal murine embryonic fibroblasts (BALB/3T3). Moreover, the antiproliferative activities of two commonly used anticancer drugs, cisplatin and doxorubicin, were also determined. The results are shown in Table 1. Detailed information concerning biological assay can be found in the Supporting Information.

**Table 1:** Antiproliferative Activity (IC_50_) (μM) and the Calculated Values of the Selectivity (SI) and Resistance (RI) Indices of MON, Its Macrocyclic Derivatives, and Reference Drugs

	cancer cells									normal cells
	A549		MCF-7		LoVo		LoVo/DX			BALB/3T3
compound	IC_50_	SI^a^	IC_50_	SI^a^	IC_50_	SI^a^	IC_50_	SI^a^	RI^b^	IC_50_
monensin, **1**	0.36 ± 0.14	6.1	0.53 ± 0.03	4.2	0.36 ± 0.11	6.1	0.17 ± 0.04	13.0	0.5	2.21 ± 0.35
**5**	7.32 ± 0.59	1.9	6.32 ± 0.79	2.2	4.46 ± 0.48	3.0	2.19 ± 0.57	6.2	0.5	13.6 ± 5.9
**6**	15.5 ± 2.9	1.5	12.2 ± 1.6	2.0	12.0 ± 2.9	2.0	4.14 ± 1.02	5.8	0.3	23.9 ± 2.7
**8**	11.2 ± 1.2	2.1	12.6 ± 3.2	1.9	7.36 ± 0.29	3.3	5.21 ± 1.15	4.6	0.7	24.0 ± 1.3
cisplatin	2.32 ± 0.90	1.3	2.42 ± 0.76	1.2	2.53 ± 0.79	1.2	2.4 ± 1.3	1.3	0.9	3.01 ± 1.75
doxorubicin	0.15 ± 0.06	0.3	0.16 ± 0.08	0.3	0.11 ± 0.03	0.5	5.9 ± 2.6	<0.1	53.6	0.05 ± 0.02

a Selectivity index (SI) was calculated as the ratio of the IC_50_ value for the normal cell line BALB/3T3 to the IC_50_ value of a respective cancer cell line. A SI greater than 1.0 identifies compounds with antiproliferative activity toward cancer cells greater than toxicity against normal cells, while the compounds with SI values greater than 3.0 are considered highly selective.^17,28,33^

b Resistance index (RI) was calculated as the ratio of the IC_50_ value for the LoVo/DX cell line to the IC_50_ value of the LoVo cell line. According to the RI value, the cell lines can be drug-sensitive (RI value in the range of 0–2), moderately drug-sensitive (RI value in the range of 2–10), and strongly drug-resistant (RI value above 10).^17,28,34^

First, the high antiproliferative activity of unmodified **MON** is worth highlighting. This ionophore proved to be effective against all tested cancer cell lines with IC_50_ values <0.6 μM (compound **1**, Table 1). These results strongly support the thesis that **MON** is one of the most antiproliferative active ionophores, displaying higher biological activity than e.g. salinomycin or lasalocid.^17^ With IC_50_ value of 2.21 μM against normal BALB/3T3 cell line, the values of selectivity indices (SI) for **MON** were within the range of 4.2–6.1 for nonresistant cell lines, and 13.0 for LoVo/DX cell line. This indicates that **MON** effectively overcomes drug resistance of the LoVo/DX cell line, with the resistance index (RI) value of 0.5 (Table 1).

Second, macrocyclic **MON** derivatives exhibit lower antiproliferative activity than the unmodified ionophore, which supports previous literature reports,^15,21,24^ however, these compounds display much lower toxicity toward the normal BALB/3T3 cell line than **MON** itself (Table 1). The derivative with the highest biological activity in this series is compound **5**, which is a macrocyclic lactone of **MON** obtained by the click reaction (Table 1). Its several-fold higher activity than that of the structurally similar compound **6** (macrocyclic lactam obtained by the click reaction) suggests that the enzymatic stability of the linkers used is of key importance in the design of ionophore-based macrocyclic compounds. As mentioned above, the relatively low physiological stability of the ester bond compared to the amide bond means that this compound may undergo hydrolysis in the intracellular environment.^27^ Therefore, the increased antiproliferative activity of compound **5** may be a consequence of the cleavage of ester bond by cellular esterases, which leads to the reformation of the pseudocyclic scaffold. Interestingly, compound **6** is more effective in overcoming drug resistance of the LoVo/DX cell line than its ester analogue **5** (RI = 0.3 for **6** versus RI = 0.5 for **5**, Table 1), and the remaining tested compounds including **MON**. Compound **8** is characterized by the lowest toxicity toward normal BALB/3T3 cell line of all tested compounds, which is why the selectivity index toward the A549 and LoVo cell lines is the highest among the macrocyclic **MON** derivatives. However, this compound shows a lower ability to overcome drug resistance of the LoVo/DX cell line than the other **MON** derivatives (Table 1).

Lastly, despite the high therapeutic potential of cisplatin and doxorubicin, particular attention should be paid to their immense toxicity toward the BALB/3T3 cell line. All obtained macrocyclic **MON** derivatives are characterized by lower toxicity and higher selectivity indices than the reference drugs, which is their indisputable advantage (Table 1). Moreover, compound **5** showed even higher antiproliferative activity against the LoVo/DX cell line than cisplatin (IC_50_ = 2.19 μM versus IC_50_ = 2.40 μM, respectively, Table 1). Taking into account the resistance index, the ability of the obtained compounds to overcome drug resistance of the LoVo/DX cancer cell line is higher than that of reference drugs used (Table 1).

## Conclusions

Herein we present a new, efficient and scalable method for obtaining C26-azide and amine of **MON**, which is the main representative of ionophore antibiotics. This discovery opens a number of synthetic pathways that can be used to obtain new, highly bioactive analogues of this compound. In this work we decided to transform the pseudocyclic scaffold of **MON** into macrocyclic products via an amidation (macrolactamization) and click reaction using Meldal protocol. All three macrocyclic derivatives demonstrate the ability to form complexes with monovalent (Li^+^, Na^+^, K^+^, Rb^+^, and Cs^+^) and divalent (Mg^2+^, Ca^2+^, Sr^2+^, and Ba^2+^) metal cations, as demonstrated by ESI-MS spectrometry. Furthermore, scXRD analysis showed that cyclic derivatives of **MON** should be recognized as ion pair receptors, as they exhibit interactions not only with cations, but also perchlorate anion which was used for crystallization and spectrometric analyses. Finally, the analysis of biological activities of all obtained analogues was performed, which showed that macrocyclic **MON** derivatives exhibit antiproliferative activity at the micromolar level toward all tested cancer cell lines. These compounds display lower activity, but also lower cytotoxicity than the unmodified ionophore, which may suggest deteriorated bioavailability of such macrocycles. Moreover, the type of linker used had a key impact on the biological activity of the obtained macrocycles, which is a good starting point for scientists who want to develop new bioactive agents based on the skeleton of pseudocyclic compounds, such as ionophore antibiotics.

## Experimental Section

### General Procedures

All reagents were purchased from two sources–Merck or Trimen Chemicals S.A., and used without further purification. Deuterated solvents for NMR analysis (CD_2_Cl_2_, CDCl_3_, and CD_3_CN) were stored over 3 Å molecular sieves for several days. Reaction mixtures were stirred using Teflon-coated magnetic stir bars and were monitored by thin layer chromatography (TLC) using aluminum-backed plates (Merck 60 F_254_). TLC plates were visualized by UV-light (254 nm), followed by treatment with phosphomolybdic acid (PMA) (5% in absolute ethanol) and gentle heating. Products of the reactions were purified using CombiFlash Rf+ Lumen Flash Chromatography System (Teledyne Isco) with integrated ELS and UV detectors. All solvents used in flash chromatography were of HPLC grade (Merck) and were used as received. Solvents were removed using a rotary evaporator.

NMR spectra were recorded on a Bruker AvanceNEO 600 (^1^H NMR at 600 MHz and ^13^C NMR at 151 MHz) magnetic resonance spectrometer. ^1^H NMR spectra are reported in chemical shifts downfield from TMS using the respective residual solvent peak as internal standard (CDCl_3_ δ 7.26 ppm; CD_2_Cl_2_ δ 5.32 ppm; CD_3_CN δ 1.94 ppm). ^1^H NMR spectra are described as follows: chemical shift (δ, ppm), multiplicity (s = singlet, d = doublet, t = triplet, p = quintet, dd = doublet of doublets, dt = doublet of triplets, td = triplet of doublets, dq = doublets of quartets, qd = quartet of doublets, ddd = doublet of doublets of doublets, dqd = doublet of quartets of doublets, m = multiplet), coupling constant(s) in Hz, and integration. Significant peaks are reported within the overlapping ∼2.00–1.20 ppm region of the ^1^H NMR spectra. ^13^C NMR spectra are reported in chemical shifts downfield from TMS using the respective residual solvent peak as internal standard (CDCl_3_ δ 77.16 ppm; CD_2_Cl_2_ δ 53.84 ppm; CD_3_CN δ 1.32 ppm, 118.26 ppm). Line broadening parameters were 0.5 or 1.0 Hz, while the error of chemical shift value was 0.1 ppm.

Electrospray ionization high-resolution mass spectra (ESI-HRMS) were recorded on a QTOF (Bruker micrOTOF-q) mass spectrometer in the positive ion detection mode. Samples were prepared in dry acetonitrile with the addition of NaClO_4_. The mass range for ESI experiments was from *m*/*z* = 400 to *m*/*z* = 1000. To analyze the ability of compounds **5**, **6** and **8** to form complexes with metal cations, a electrospray ionization (ESI) measurements with positive mode detection was performed using PurIonMass Spectrometer Model L. The complexes of compound **5**, **6** and **8** with cations were prepared by mixing acetonitrile solutions of these compounds and respective cation salts in the 1:5 mixture of acetonitrile. The concentration of studied compounds in final solutions used for the ESI measurement was 5 × 10^–5^ mol dm^–3^. The samples were infused directly to the ESI source. The major parameters were set as follows: Source: ESI + 3.5 kV 350 °C, Capillary: 150 V 300 °C Offset: 25 V Span: 0 V, full scan ranging 1000–2000 (*m*/*z*).

### Synthesis

#### Isolation of MON

The sodium salt of **MON** was isolated from commercially available veterinary premix–Coxidin, as previously described.^21^ The obtained sodium salt of **MON** was then extracted with a solution of sulfuric acid (pH = 1), giving **MON** ready for further synthesis.

#### Synthesis of Monensin Azide (Compound **2**)

To a solution of **MON** (11.0 g, 16.39 mmol, 1.0 equiv) in toluene (120 mL) upon stirring, a solution of 0.1 M Na_2_CO_3_ was added (150 mL). After that, tosyl chloride (9.37 g, 49.18 mmol, 3.0 equiv) was added to the resulting mixture in one portion. The resulting solution was stirred at room temperature for the next 72 h. The reaction mixture was then transferred to a separatory funnel and extracted with 0.1 M Na_2_CO_3_ solution and brine. After that, the organic layer was concentrated under reduced pressure, and directed subsequently to the next stage of the process. The excess of sodium azide (3.19 g, 49.09 mmol, 3.0 equiv) was dissolved in 30 mL of DMSO (complete conversion of **MON** to tosylate was assumed). The solution of crude tosylate dissolved in 20 mL of DMSO was then added to the mixture. The resulting solution was stirred at room temperature for the next 72 h. The mixture was then diluted with large amount of water (over 300 mL) and extracted several times with methylene chloride. This step can be dangerous due to the possibility of an explosion as a result of the reaction between sodium azide and the chlorinated solvent, so the sequence of operations must be strictly followed.^35^ Alternatively, compound **2** can be extracted from the aqueous phase into the ethyl acetate phase. Purification on silica gel using the CombiFlash system (0 → 40% EtOAc/*n*-hexane) gave the pure product as a clear oil. The oil was diluted in *n*-pentane and evaporated to dryness three times to form an amorphous solid, with an isolated yield of 62% (7.10 g), a single spot by TLC; stains green with PMA; ^13^C NMR{^1^H} (151 MHz, CDCl_3_) δ 176.3, 107.7, 99.1, 86.0, 85.7, 84.6, 83.0, 81.8, 76.4, 75.6, 71.1, 67.0, 58.0, 56.1, 41.0, 38.5, 36.8, 36.0, 34.7, 34.3, 34.1, 33.4, 33.0, 32.6, 31.3, 30.6, 27.5, 27.3, 17.5, 16.1, 15.8, 15.1, 10.8, 10.6, 8.3 ppm; ^1^H NMR (600 MHz, CDCl_3_) δ 5.79 (s, 1H), 4.60 (s, 1H), 4.32 (ddd, *J* = 10.4, 6.0, 2.9 Hz, 1H), 4.10 (dd, *J* = 11.3, 2.2 Hz, 1H), 4.08 (d, *J* = 4.0 Hz, 1H), 4.07 (dd, *J* = 10.2, 3.4 Hz, 1H), 3.78 (s, 1H), 3.49 (d, *J* = 13.1 Hz, 1H), 3.46 (dd, *J* = 10.5, 5.1 Hz, 1H), 3.36 (s, 3H), 3.24 (dd, *J* = 9.6, 2.5 Hz, 1H), 3.07 (d, *J* = 13.1 Hz, 1H), 2.63 (dq, *J* = 9.5, 6.7 Hz, 1H), 2.28 (td, *J* = 11.3, 6.3 Hz, 1H), 2.24–2.18 (m, 2H), 2.15 (dqd, *J* = 13.7, 6.9, 2.5 Hz, 1H), 2.04–1.93 (m, 3H), 1.91–1.80 (m, 2H), 1.74–1.31 (m, 13H), 1.48 (s, 3H), 1.24 (d, *J* = 6.7 Hz, 3H), 1.09 (d, *J* = 6.9 Hz, 3H), 0.90 (d, *J* = 7.0 Hz, 3H), 0.89 (m, 3H), 0.88 (d, *J* = 7.2 Hz, 3H), 0.86 (dd, *J* = 6.6, 2.2 Hz, 6H) ppm; MS (ESI) *m*/*z*: [M + Na]^+^ Calcd for C_36_H_61_N_3_NaO_10_ 718.4249; Found 718.4242.

#### Synthesis of Propargyl Ester of MON Azide (Compound **3**)

Compound **2** (1.0 g, 1.44 mmol, 1.0 equiv) was dissolved in toluene (25 mL) and the solution was heated to 90 °C on a heating mantle. After 15 min DBU (372 mg, 2.44 mmol, 1.7 equiv) was added dropwise to the reaction mixture, and after another 20 min, propargyl bromide (513 mg, 4.31 mmol, 3.0 equiv) was added dropwise. The solution changed its color to brownish after a few minutes. After 24 h, the reaction mixture was concentrated on a rotary evaporator. Purification on silica gel using the CombiFlash system (0% → 20% EtOAc/*n*-hexane) gave the pure ester **3** as a clear oil. After twice evaporation to dryness with *n*-pentane, the product remained in an oily form (886 mg, 84% yield), a single spot by TLC; stains green with PMA; ^13^C{^1^H} NMR (151 MHz, CD_3_CN) δ 175.2, 108.4, 99.0, 87.9, 86.9, 86.4, 84.1, 82.3, 79.1, 77.9, 77.6, 76.5, 71.9, 68.4, 58.7, 57.5, 52.8, 41.4, 39.8, 37.9, 37.4, 36.9, 35.6, 35.2, 35.0, 34.7, 34.1, 32.7, 32.2, 31.0, 29.1, 26.7, 17.9, 16.4, 16.3, 12.8, 12.4, 11.3, 8.6 ppm; ^1^H NMR (600 MHz, CD_3_CN) δ 4.75 (dd, *J* = 15.8, 2.5 Hz, 1H), 4.69 (dd, *J* = 15.8, 2.5 Hz, 1H), 4.51 (s, 1H), 4.23 (ddd, *J* = 8.7, 6.3, 2.9 Hz, 2H), 3.99 (dd, *J* = 9.1, 2.4 Hz, 1H), 3.94 (d, *J* = 4.2 Hz, 1H), 3.72–3.68 (m, 2H), 3.60 (dd, *J* = 10.3, 5.2 Hz, 1H), 3.51 (m, 1H), 3.33 (s, 3H), 3.28 (d, *J* = 12.6 Hz, 1H), 3.06 (d, *J* = 12.6 Hz, 1H), 2.87 (t, *J* = 2.5 Hz, 1H), 2.67 (qd, *J* = 6.9, 5.5 Hz, 1H), 2.31–2.17 (m, 3H), 2.14 (ddd, *J* = 11.8, 9.2, 2.3 Hz, 1H), 2.04–1.27 (m, 17H), 1.37 (s, 3H), 1.17 (d, *J* = 6.9 Hz, 3H), 0.98 (d, *J* = 6.9 Hz, 3H), 0.93 (d, *J* = 7.0 Hz, 3H), 0.92–0.88 (m, 9H), 0.82 (d, *J* = 6.8 Hz, 3H) ppm; MS (ESI) *m*/*z*: [M + Na]^+^ Calcd for C_39_H_63_N_3_NaO_10_ 756.4406; Found 756.4396.

#### Synthesis of Propargyl Amide of MON Azide (Compound **4**)

Upon stirring to a solution of **2** (1.0 g, 1.44 mmol, 1.0 equiv) in CH_2_Cl_2_ (25 mL), cooled in an ice bath, DCC (356 mg, 1.72 mmol, 1.2 equiv) was added in one portion. After 20 min HOBt (97 mg, 0.72 mmol, 0.5 equiv) was added. After another 10 min, propargylamine (198 mg, 3.69 mmol, 2.5 equiv) was added dropwise to the reaction mixture. After 24 h, the resulting DCU precipitate was filtered off and the reaction mixture was concentrated under reduced pressure. Purification on silica gel using the CombiFlash system (0 → 30% EtOAc/*n*-hexane) gave the pure product as a clear oil. After twice evaporation to dryness with *n*-pentane, the product remained in an oily form (927 mg, 88% yield), a single spot by TLC; stains green with PMA; ^13^C{^1^H} NMR (151 MHz, CD_3_CN) δ 175.1, 108.3, 99.1, 87.6, 86.8, 86.4, 84.1, 82.6, 81.7, 77.9, 77.5, 72.3, 71.9, 68.3, 58.6, 57.3, 42.7, 39.8, 38.0, 37.4, 36.4, 35.7, 35.1, 35.0, 34.6, 34.0, 32.9, 32.2, 31.0, 29.2, 29.1, 26.8, 17.9, 16.4, 16.3, 14.7, 12.4, 11.2, 8.60 ppm; ^1^H NMR (600 MHz, CD_3_CN) δ 6.82 (s, 1H), 4.70 (s, 1H), 4.24 (ddd, *J* = 9.5, 6.3, 3.2 Hz, 1H), 4.17 (d, *J* = 8.7 Hz, 1H), 4.00–3.91 (m, 4H), 3.73 (dd, *J* = 10.2, 3.5 Hz, 1H), 3.68–3.65 (m, 1H), 3.58 (dd, *J* = 10.3, 5.1 Hz, 1H), 3.38–3.31 (m, 2H), 3.34 (s, 3H), 3.06 (d, *J* = 12.6 Hz, 1H), 2.57 (s, 1H), 2.47–2.41 (m, 1H), 2.31–2.24 (m, 3H), 2.21 (dd, *J* = 11.2, 6.3 Hz, 1H), 2.16–2.11 (m, 1H), 2.04–1.26 (m, 16H), 1.38 (s, 3H), 1.12 (d, *J* = 6.9 Hz, 3H), 0.99 (d, *J* = 6.9 Hz, 3H), 0.93 (d, *J* = 7.0 Hz, 3H), 0.90 (t, *J* = 7.4 Hz, 3H), 0.89 (d, *J* = 6.3 Hz, 3H), 0.88 (d, *J* = 7.1 Hz, 3H), 0.83 (d, *J* = 6.8 Hz, 3H) ppm; MS (ESI) *m*/*z*: [M + Na]^+^ Calcd for C_39_H_64_N_4_NaO_9_ 755.4566; Found 755.4554.

#### Synthesis of Macrocyclic Lactone of MON Obtained by Click Reaction (Compound **5**)

Under a nitrogen atmosphere, to a solution of **3** (500 mg, 0.68 mmol, 1.0 equiv) in anhydrous CH_3_CN, DIPEA (264 mg, 2.04 mmol, 3.0 equiv) was introduced, followed by the addition of catalytic CuI (13 mg, 0.068 mmol, 0.1 equiv) in one portion. The reaction mixture was stirred at room temperature for 24 h. After the consumption of the substrate (TLC and ESI-MS control), the organic solvent was removed on a rotary evaporator. The oily residue was dissolved in a small amount of CH_2_Cl_2_ and extracted a few times with 10% aq. EDTA solution. Organic phases were concentrated under reduced pressure. Purification on silica gel using the CombiFlash system (0 → 40% EtOAc/*n*-hexane) gave the pure product as a clear oil. The oil was diluted in *n*-pentane and evaporated to dryness three times to form an amorphous solid, with an isolated yield of 74% (370 mg), a single spot by TLC; stains green with PMA; ^13^C{^1^H} NMR (151 MHz, CD_2_Cl_2_) δ 175.5, 142.9, 125.0, 108.1, 96.9, 86.5, 86.3, 85.8, 85.2, 79.9, 78.0, 76.3, 71.5, 67.7, 59.4, 58.2, 57.8, 41.2, 38.7, 38.4, 37.5, 37.3, 35.6, 34.4, 33.8, 33.5, 32.5, 30.1, 29.7, 27.5, 23.1, 17.8, 16.7, 16.3, 14.3, 13.5, 11.3, 11.1, 8.2 ppm; ^1^H NMR (600 MHz, CD_2_Cl_2_) δ 7.90 (s, 1H), 5.27 (d, *J* = 13.1 Hz, 1H), 5.15 (d, *J* = 13.1 Hz, 1H), 4.91 (s, 1H), 4.60 (d, *J* = 14.0 Hz, 1H), 4.38 (d, *J* = 8.4 Hz, 1H), 4.26 (d, *J* = 13.9 Hz, 1H), 4.24–4.20 (m, 1H), 3.88 (dd, *J* = 10.7, 2.1 Hz, 1H), 3.82 (d, *J* = 5.5 Hz, 1H), 3.67–3.62 (m, 2H), 3.57–3.53 (m, 1H), 3.47 (dd, *J* = 4.1, 2.6 Hz, 1H), 3.33 (s, 3H), 2.62 (qd, *J* = 7.0, 4.5 Hz, 1H), 2.35 (ddd, *J* = 10.1, 7.2, 3.4 Hz, 1H), 2.29 (s, 1H), 2.16–2.08 (m, 2H), 2.05 (ddd, *J* = 11.5, 5.6, 2.7 Hz, 1H), 1.97 (ddd, *J* = 9.3, 8.8, 2.8 Hz, 2H), 1.82 (dt, *J* = 11.4, 8.9 Hz, 1H), 1.76–1.31 (m, 10H), 1.45 (s, 3H), 1.26 (d, *J* = 6.9 Hz, 3H), 1.25 (s, 3H), 1.04 (d, *J* = 7.2 Hz, 3H), 0.99 (d, *J* = 7.0 Hz, 3H), 0.97 (d, *J* = 6.7 Hz, 3H), 0.91 (t, *J* = 7.4 Hz, 3H), 0.87–0.85 (m, 6H) ppm; MS (ESI) *m*/*z*: [M + Na]^+^ Calcd for C_39_H_63_N_3_NaO_10_ 756.4406; Found 756.4394.

#### Synthesis of Macrocyclic Lactam of MON Obtained by Click Reaction (Compound **6**)

Under a nitrogen atmosphere, to a solution of **4** (500 mg, 0.68 mmol, 1.0 equiv) in anhydrous CH_3_CN, DIPEA (264 mg, 2.04 mmol, 3.0 equiv) was introduced, followed by the addition of catalytic CuI (13 mg, 0.068 mmol, 0.1 equiv) in one portion. The reaction mixture was stirred at room temperature for 24 h. After the consumption of the substrate (TLC and ESI-MS control), the organic solvent was removed on a rotary evaporator. The oily residue was dissolved in a small amount of CH_2_Cl_2_ and extracted a few times with 10% aq. EDTA solution. Organic phases were concentrated under reduced pressure. Purification on silica gel using the CombiFlash system (0 → 30% EtOAc/*n*-hexane) gave the pure product as a clear oil. The oil was diluted in *n*-pentane and evaporated to dryness three times to form an amorphous solid, with an isolated yield of 52% (260 mg), a single spot by TLC; stains green with PMA; ^13^C{^1^H} NMR (151 MHz, CD_2_Cl_2_) δ 176.7, 144.9, 128.3, 106.7, 97.6, 87.2, 85.8, 85.2, 83.6, 79.4, 78.2, 76.1, 70.4, 68.7, 57.4, 57.3, 43.3, 39.5, 39.1, 37.8, 36.2, 35.5, 34.9, 34.6, 34.0, 33.7, 33.3, 32.5, 31.7, 30.9, 28.6, 27.9, 17.4, 16.5, 16.3, 16.3, 12.5, 10.7, 8.4 ppm; ^1^H NMR (600 MHz, CD_2_Cl_2_) δ 7.84 (s, 1H), 6.52–6.48 (m, 1H), 4.58 (s, 1H), 4.56 (d, *J* = 14.4 Hz, 1H), 4.45–4.37 (m, 3H), 4.31 (dd, *J* = 14.9, 5.9 Hz, 1H), 4.23 (d, *J* = 4.5 Hz, 1H), 3.87–3.85 (m, 1H), 3.75 (dd, *J* = 10.0, 2.2 Hz, 1H), 3.63 (dd, *J* = 10.6, 1.8 Hz, 1H), 3.55 (dd, *J* = 11.3, 4.1 Hz, 1H), 3.40 (dd, *J* = 8.0, 2.7 Hz, 1H), 3.31 (s, 3H), 3.28 (s, 1H), 2.62 (p, *J* = 7.0 Hz, 1H), 2.46–2.37 (m, 3H), 2.24 (dd, *J* = 11.8, 10.0 Hz, 1H), 2.03–1.39 (m, 17H), 1.62 (s, 3H), 1.24 (d, *J* = 7.0 Hz, 3H), 1.02 (dd, *J* = 14.1, 7.0 Hz, 9H), 0.98 (t, *J* = 7.5 Hz, 3H), 0.90 (d, *J* = 6.1 Hz, 3H), 0.85 (d, *J* = 7.1 Hz, 3H) ppm; MS (ESI) *m*/*z*: [M + Na]^+^ Calcd for C_39_H_64_N_4_NaO_9_ 755.4566; Found 755.4553.

#### Synthesis of Monensin Amine (Compound **7**)

Under a nitrogen atmosphere, to a solution of **2** (2.0 g, 2.87 mmol, 1.0 equiv) in methanol upon stirring, the catalytic amount of palladium on carbon (Pd/C) was added. Then, a hydrogen-filled balloon was connected to the system. The reaction was continued until the azide was completely consumed (TLC and ESI-MS control), replacing the hydrogen balloon if necessary (typically 72 h). After this time, the reaction mixture was filtered through Celite to remove Pd/C, and concentrated under reduced pressure. Purification on silica gel using the CombiFlash system (0 → 30% MeOH/CHCl_3_) gave the pure product as a clear oil. The oil was diluted in *n*-pentane and evaporated to dryness three times to form an amorphous solid, with an isolated yield of 63% (1.22 g), a single spot by TLC; stains green with PMA; ^13^C{^1^H} NMR (151 MHz, CD_2_Cl_2_) δ 181.1, 108.0, 95.8, 86.4, 85.5, 84.2, 82.5, 77.1, 75.2, 71.9, 68.0, 58.0, 47.4, 44.4, 38.7, 37.8, 37.3, 36.7, 35.1, 34.5, 34.3, 34.0, 32.8, 31.5, 31.1, 30.1, 28.1, 28.0, 17.6, 16.7, 16.2, 15.9, 11.2, 10.8, 8.6 ppm; ^1^H NMR (600 MHz, CD_2_Cl_2_) δ 6.17 (s, 7H hydrate), 4.33–4.29 (m, 1H), 4.09 (d, *J* = 11.2 Hz, 1H), 3.94 (d, *J* = 3.5 Hz, 1H), 3.88 (dd, *J* = 10.6, 2.5 Hz, 1H), 3.80 (d, *J* = 2.6 Hz, 1H), 3.45 (dd, *J* = 10.9, 4.0 Hz, 1H), 3.35–3.30 (m, 2H), 3.33 (s, 3H), 3.16 (d, *J* = 8.9 Hz, 1H), 3.08 (d, *J* = 11.8 Hz, 1H), 2.70 (d, *J* = 11.9 Hz, 1H), 2.39 (td, *J* = 13.5, 6.7 Hz, 1H), 2.22–2.12 (m, 5H), 2.09–2.02 (m, 2H), 1.99–1.22 (m, 16H), 1.46 (s, 3H), 1.16 (d, *J* = 6.7 Hz, 3H), 1.04 (d, *J* = 6.8 Hz, 3H), 0.93 (t, *J* = 7.4 Hz, 3H), 0.90 (d, *J* = 7.0 Hz, 3H), 0.88 (d, *J* = 3.6 Hz, 3H), 0.87 (d, *J* = 2.8 Hz, 3H), 0.84 (d, *J* = 6.5 Hz, 3H) ppm; MS (ESI) *m*/*z*: [M + H]^+^ Calcd for C_36_H_64_NO_10_ 670.4525 Found 670.4519; [M + Na]^+^ Calcd for C_36_H_63_NNaO_10_ 692.4344 Found 692.4332.

#### Synthesis of Macrocyclic Lactam of MON (Compound **8**)

##### Method A

Upon stirring to a solution of **7** (1.0 g, 1.49 mmol, 1.0 equiv) in DMF (15 mL) DIPEA (1.16 g, 8.96 mmol, 6.0 equiv) was added in one portion. After 15 min HATU (0.738 g, 1.94 mmol, 1.3 equiv) was added to the mixture. The reaction was carried out for 24 h and then concentrated under reduced pressure, and extracted with 0.1 M Na_2_CO_3_ solution and water. Purification on silica gel using the CombiFlash system (0 → 100% EtOAc/*n*-hexane) gave the pure product as a clear oil. The oil was diluted in *n*-pentane and evaporated to dryness three times to form an amorphous solid, with an isolated yield of 72% (701 mg), a single spot by TLC; stains green with PMA.

##### Method B

Upon stirring to a solution of **7** (1.0 g, 1.49 mmol, 1.0 equiv) in DMF (15 mL) DIPEA (1.16 g, 8.96 mmol, 6.0 equiv) was added in one portion. After 15 min HBTU (0.736 g, 1.94 mmol, 1.3 equiv) was added to the mixture. The reaction was carried out for 24 h and then concentrated under reduced pressure, and extracted with 0.1 M Na_2_CO_3_ solution and water. Purification on silica gel using the CombiFlash system (0 → 100% EtOAc/*n*-hexane) gave the pure product as a clear oil. The oil was diluted in *n*-pentane and evaporated to dryness three times to form an amorphous solid, with an isolated yield of 10% (97 mg), a single spot by TLC; stains green with PMA.

##### Method C

Upon stirring to a solution of **7** (250 mg, 0.37 mmol, 1.0 equiv) in CH_2_Cl_2_ (15 mL) cooled in an ice bath, DCC (100 mg, 0.48 mmol, 1.3 equiv) was added in one portion. After 24 h, the resulting DCU precipitate was filtered off and the reaction mixture was concentrated under reduced pressure. Purification on silica gel using the CombiFlash system (0 → 100% EtOAc/*n*-hexane) gave the pure product as a clear oil. The oil was diluted in *n*-pentane and evaporated to dryness three times to form an amorphous solid, with an isolated yield of 16% (39 mg), a single spot by TLC; stains green with PMA.

##### Method D

Upon stirring to a solution of **7** (250 mg, 0.37 mmol, 1.0 equiv) in CH_2_Cl_2_ (15 mL) cooled in an ice bath, EDCI (75 mg, 0.48 mmol, 1.3 equiv) was added in one portion. After 24 h, no product formation was observed (TLC and ESI-MS control). Increasing the reaction time to 72 h did not increase its efficiency. Nevertheless, purification on silica gel using the CombiFlash system (0 → 100% EtOAc/*n*-hexane) was performed. The reaction product was not isolated.

^13^C{^1^H} NMR (151 MHz, CD_3_CN) δ 178.3 109.4, 98.6, 87.6, 86.4, 86.1, 83.5, 78.1, 74.5, 72.4, 69.2, 58.6, 46.4, 41.9, 39.1, 38.8, 38.7, 37.9, 37.2, 36.7, 36.1, 35.0, 34.4, 33.6, 33.3, 32.0, 31.5, 28.4, 25.6, 17.9, 17.1, 15.9, 15.3, 11.1, 10.3, 8.8 ppm; ^1^H NMR (600 MHz, CD_3_CN) δ 7.41 (s, 1H), 5.12 (s, 1H), 4.65 (d, *J* = 10.2 Hz, 1H), 4.32–4.25 (m, 2H), 4.15 (d, *J* = 4.7 Hz, 1H), 4.10 (dd, *J* = 13.4, 10.3 Hz, 1H), 3.86 (dd, *J* = 9.8, 2.8 Hz, 1H), 3.72–3.68 (m, 1H), 3.66 (dd, *J* = 11.1, 4.6 Hz, 1H), 3.40 (s, 3H), 3.35 (t, *J* = 2.7 Hz, 1H), 2.84 (s, 3H), 2.69 (qd, *J* = 7.1, 2.7 Hz, 1H), 2.49 (dd, *J* = 13.5, 1.6 Hz, 1H), 2.37–2.31 (m, 1H), 2.23–2.16 (m, 2H), 2.09–2.00 (m, 2H), 1.96–1.91 (m, 2H), 1.81–1.27 (m, 11H), 1.45 (s, 3H), 1.08 (d, *J* = 7.2 Hz, 3H), 0.98 (d, *J* = 7.1 Hz, 3H), 0.97 (t, *J* = 7.4 Hz, 3H), 0.93 (d, *J* = 6.7 Hz, 3H), 0.89 (dd, *J* = 6.9, 0.8 Hz, 6H), 0.83 (d, *J* = 6.1 Hz, 3H) ppm; MS (ESI) *m*/*z*: [M + Na]^+^ Calcd for C_36_H_61_NNaO_9_ 674.4239; Found 674.4228.

### Anticancer Activity Studies

Stock solutions of all tested compounds in a concentration of 10 mg/mL were prepared ex tempore for each experiment by dissolving 1 mg of the respective compound in 100 μL of dimethyl sulfoxide (DMSO, Avantor Performance Materials Poland, Gliwice, Poland). The solvent for further dilutions was OR culture medium, which is a mixture of OptiMEM and Roswell Park Memorial Institute (RPMI) 1640 medium (1:1) (OptiMEM–GIBCO, Thermo Fisher Scientific, USA, RPMI with l-glutamine–IIET, Wroclaw, Poland), supplemented with 5% fetal bovine serum (FBS, HyClone, Cytiva, USA). The compounds were tested at 8 different concentrations from the range of 100–0.001 μg/mL. As a control, cisplatin and doxorubicin were used in the concentration range of 10–0.01 μg/mL (both drugs from Accord Healthcare, Warsaw, Poland), as well as DMSO solvent in concentrations corresponding to its concentration in the samples, from the range of 1–0.001%.

### Cell Lines and Culturing Conditions

Four human cancer cell lines and one murine normal cell line were used to evaluate antiproliferative activity of monensin and its derivatives: human lung carcinoma (A549), human breast adenocarcinoma (MCF7), human colon adenocarcinoma cell lines sensitive and resistant to doxorubicin (LoVo) and (LoVo/DX), respectively, and normal murine embryonic fibroblast cell line (BALB/3T3) clone A31. All cell lines are available in the IIET cell line bank (Wroclaw, Poland). A549 cells were cultured in a mixture of OptiMEM and Roswell Park Memorial Institute (RPMI) 1640 medium (1:1) (OptiMEM–GIBCO, Thermo Fisher Scientific, USA, RPMI with l-glutamine–IIET, Wroclaw, Poland), supplemented with 10% fetal bovine serum (FBS, HyClone, Cytiva, USA). MCF7 cells were cultured in a mixture of Eagle’s medium (IIET, Wroclaw, Poland), supplemented with 10% fetal bovine serum, 2 mM l-glutamine, 1% MEM endogenous amino acid solution (Sigma-Aldrich, Germany), and 8 μg/mL insulin from bovine pancreas (Sigma-Aldrich Chemie GmbH, Steinheim, Germany). LoVo and LoVo/DX cells were cultured in OR medium, supplemented with 10% fetal bovine serum (FBS, HyClone, Cytiva, USA) and 1.0 mM of sodium pyruvate (Sigma-Aldrich Chemie GmbH, Steinheim, Germany). Additionally, the medium for LoVo/DX contained 0.1 μg/mL doxorubicin. BALB/3T3 fibroblasts were cultured in Dulbecco’s medium, supplemented with 10% fetal bovine serum (HyClone) and 2 mM l-glutamine. All culture media contained antibiotics: 100 U/mL penicillin and 100 μg/mL streptomycin. All cell lines were cultured in humid atmosphere at 37 °C and 5% CO_2_.

### Cell Viability Assays and SRB

Antiproliferative assays were performed using five cell lines: A549, LoVo, LoVo/DX, MCF7, and BALB/3T3. The cells were plated in 384-well plates (Greiner Bio-One, Kremsmünster, Austria) in the number of: 1500 cells per well for the A549, LoVo/DX and MCF7 cell lines and 2000 cells per well for the LoVo and BALB/3T3 cell lines. After 24 h, solutions of the tested compounds were added. The in vitro cytotoxicity of the tested compounds was determined using the sulforhodamine B (SRB) assay 2. Cultured cells were exposed to 8 different concentrations of the tested compounds from the range of 0.1–100, 0.01–10, or 0.001–1 μg/mL (depending on the activity of the compound). After 72 h of incubation, the cells were fixed by adding cold 50% (w/v) trichloroacetic acid (TCA) (Avantor Performance Materials, Gliwice, Poland) and were incubated at room temperature for 1 h. Then, the wells were washed with distilled water, and strained with 0.4% (w/v) solution of sulforhodamine B (Sigma-Aldrich, Germany) in 1% (v/v) acetic acid (Avantor Performance Materials, Gliwice, Poland) for 0.5 h. After incubation time, the plates with stained cells were rinsed with 1% (v/v) acetic acid. The protein-bound dye was solubilized with 10 mM TRIS solution (Avantor Performance Materials, Gliwice, Poland) for 0.5 h. All steps were performed at room temperature using an EL406 microplate washer (BioTek Instruments Inc., Winooski, Vermont, USA). Absorbance of each solution was read at Synergy H4 Hybrid Multi-Mode Microplate Reader (BioTek Instruments Inc., Winooski, Vermont, USA) at the 540 nm wavelength. In each experiment, samples containing specific concentrations of the respective compounds were applied in triplicate. The experiments were repeated four or five times. Inhibition of proliferation was determined using the following formula:
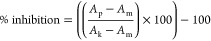


*A*_m_–absorbance of the control medium

*A*_k_–absorbance of control cells

*A*_p_–absorbance of cells treated with the tested compounds

The experimental results are presented in the form of IC_50_ ± SD (dose causing inhibition of proliferation of 50% of the cancer cell population ± standard deviation). The DMSO was inactive.

## Data Availability

The data underlying this study are available in the published article and its Supporting Information.

## Supporting Information Available

Experimental details, materials and methods, NMR, FT-IR, and ESI-MS spectra, as well as the details of X-ray crystallography of the reported compounds (PDF)
FAIR data, including the primary NMR FID files, for compounds **2**–**8** (ZIP)

## Abbreviations

A549: human lung carcinoma
aq: aqueous solution
BALB/3T3: normal murine embryonic fibroblasts
CuAAC: Cu(I)-catalyzed azide–alkyne cycloaddition
DBU: 1,8-diazabicyclo[5.4.0]undec-7-ene
DCC: *N*,*N′*-dicyclohexylcarbodiimide
DIPEA: *N*,*N*-diisopropylethylamine
DMF: dimethylformamide
DMSO: dimethyl sulfoxide
EDCI: 1-ethyl-3-(3-(dimethylamino)propyl)carbodiimide
equiv.: equivalent
ESI-MS: electrospray ionization mass spectrometry
ESI-HRMS: electrospray ionization high resolution mass spectrometry
FT-IR: Fourier-transform infrared spectroscopy
HATU: hexafluorophosphate azabenzotriazole tetramethyl uranium
HBTU: hexafluorophosphate benzotriazole tetramethyl uranium
HOBt: hydroxybenzotriazole
IC_50_: half maximal inhibitory concentration
LoVo: human colon adenocarcinoma
LoVo/DX: doxorubicin-resistant subline of LoVo
MCF-7: human breast adenocarcinoma
MON: monensin
NMR: nuclear magnetic resonance spectroscopy
RI: resistance index
rt: room temperature
SAR: structure–activity relationship
scXRD: single crystal X-ray diffraction
SI: selectivity index
TsCl: 4-toluenesulfonyl chloride
