# Supplementary material for: From Pseudocyclic to Macrocyclic Ionophores: Strategies toward the Synthesis of Cyclic Monensin Derivatives
Source: J Org Chem. 2025 Jan 10;90(3):1344–53. doi: 10.1021/acs.joc.4c02715 (PMC11773414; doi:10.1021/acs.joc.4c02715)
Supplement: Supplementary file 1 — jo4c02715_si_001.pdf [file jo4c02715_si_001.pdf]

Supplementary material

From pseudo-cyclic to macrocyclic ionophores: strategies towards the synthesis of cyclic monensin derivatives

Michał Sulik [a], Robert Graniczny [a], Jan Janczak [b], Dagmara Kłopotowska [c] Joanna Wietrzyk [c], Adam Huczyński [a,*]

[a] *Department of Medical Chemistry, Faculty of Chemistry, Adam Mickiewicz University, Uniwersytetu Poznańskiego 8, 61–614 Poznań, Poland*

[b] *Hirszfeld Institute of Immunology and Experimental Therapy, Polish Academy of Sciences, Rudolfa Weigla 12, 53–114 Wrocław, Poland*

[c] *Institute of Low Temperature and Structure Research, Polish Academy of Sciences, Okólna 2, Wrocław, 50–422, Poland*

E-mail: adhucz@amu.edu.pl (A. Huczyński)

Index

Spectroscopic and spectrometric analysis of newly synthesized compounds S2

X-ray measurements S23

Literature S28

Spectroscopic and spectrometric analysis of newly synthesized compounds

List of spectra

Figure S1. The ^{13}C NMR spectrum of **2** in chloroform-d. S3

Figure S2. The ^{1}H NMR spectrum of **2** in chloroform-d. S3

Figure S3. The ^{13}C NMR spectrum of **3** in acetonitrile-d3. S4

Figure S4. The ^{1}H NMR spectrum of **3** in acetonitrile-d3. S4

Figure S5. The ^{13}C NMR spectrum of **4** in acetonitrile-d3. S5

Figure S6. The ^{1}H NMR spectrum of **4** in acetonitrile-d3. S5

Figure S7. The ^{13}C NMR spectrum of **5** in dichloromethane-d2. S6

Figure S8. The ^{1}H NMR spectrum of **5** in dichloromethane-d2. S6

Figure S9. The ^{13}C NMR spectrum of **6** in dichloromethane-d2. S7

Figure S10. The ^{1}H NMR spectrum of **6** in dichloromethane-d2. S7

Figure S11. The ^{13}C NMR spectrum of **7** in dichloromethane-d2. S8

Figure S12. The ^{1}H NMR spectrum of **7** in dichloromethane-d2. S8

Figure S13. The ^{13}C NMR spectrum of **8** in acetonitrile-d3. S9

Figure S14. The ^{1}H NMR spectrum of **8** in acetonitrile-d3. S9

Figure S15. The ESI mass spectra of a mixture of **5** with (a) $LiClO_4$, (b) $NaClO_4$, and (c) $KClO_4$. S10

Figure S16. The ESI mass spectra of a mixture of **5** with (a) $RbClO_4$, (b) $CsClO_4$, and (c) $Mg(ClO_4)_2$. S11

Figure S17. The ESI mass spectra of a mixture of **5** with (a) $Ca(ClO_4)_2$, (b) $Sr(ClO_4)_2$, and (c) $Ba(ClO_4)_2$. S12

Figure S18. The ESI mass spectra of a mixture of **6** with (a) $LiClO_4$, (b) $NaClO_4$, and (c) $KClO_4$. S13

Figure S19. The ESI mass spectra of a mixture of **6** with (a) $RbClO_4$, (b) $CsClO_4$, and (c) $Mg(ClO_4)_2$. S14

Figure S20. The ESI mass spectra of a mixture of **6** with (a) $Ca(ClO_4)_2$, (b) $Sr(ClO_4)_2$, and (c) $Ba(ClO_4)_2$. S15

Figure S21. The ESI mass spectra of a mixture of **8** with (a) $LiClO_4$, (b) $NaClO_4$, and (c) $KClO_4$. S16

Figure S22. The ESI mass spectra of a mixture of **8** with (a) $RbClO_4$, (b) $CsClO_4$, and (c) $Mg(ClO_4)_2$. S17

Figure S23. The ESI mass spectra of a mixture of **8** with (a) $Ca(ClO_4)_2$, (b) $Sr(ClO_4)_2$, and (c) $Ba(ClO_4)_2$. S18

Figure S24. The FT-IR spectrum of **3** in the range of 4000-500 cm^{-1}. S19

Figure S25. The FT-IR spectrum of **4** in the range of 4000-500 cm^{-1}. S19

Figure S26. The FT-IR spectrum of **5** in the range of 4000-500 cm^{-1}. S20

Figure S27. The FT-IR spectrum of **5** with the addition of $NaClO_4$ in the range of 4000-500 cm^{-1}. S20

Figure S28. The FT-IR spectrum of **6** in the range of 4000-500 cm^{-1}. S21

Figure S29. The FT-IR spectrum of **6** with the addition of $NaClO_4$ in the range of 4000-500 cm^{-1}. S21

Figure S30. The FT-IR spectrum of **8** in the range of 4000-500 cm^{-1}. S22

Figure S31. The FT-IR spectrum of **8** with the addition of $NaClO_4$ in the range of 4000-500 cm^{-1}. S22

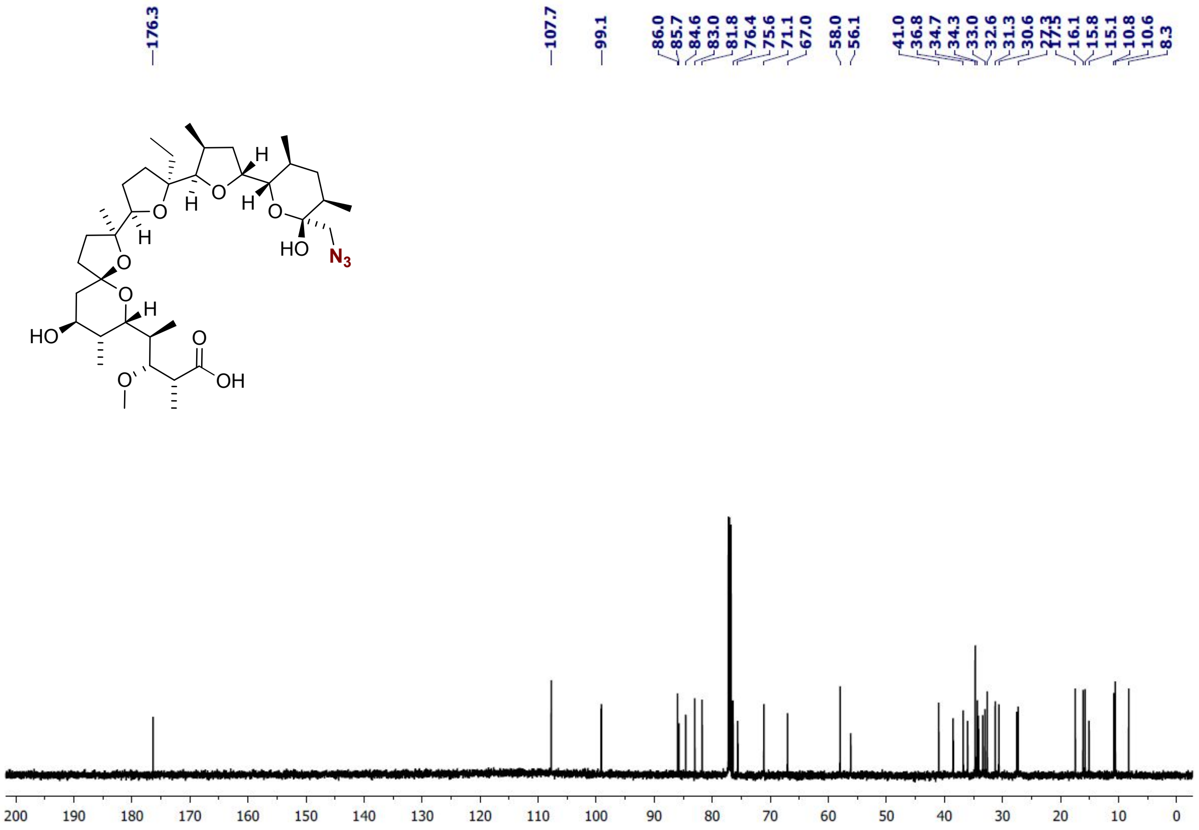

Figure S1. The $^{13}C\{^{1}H\}$ NMR (151 MHz) spectrum of **2** in chloroform-d.

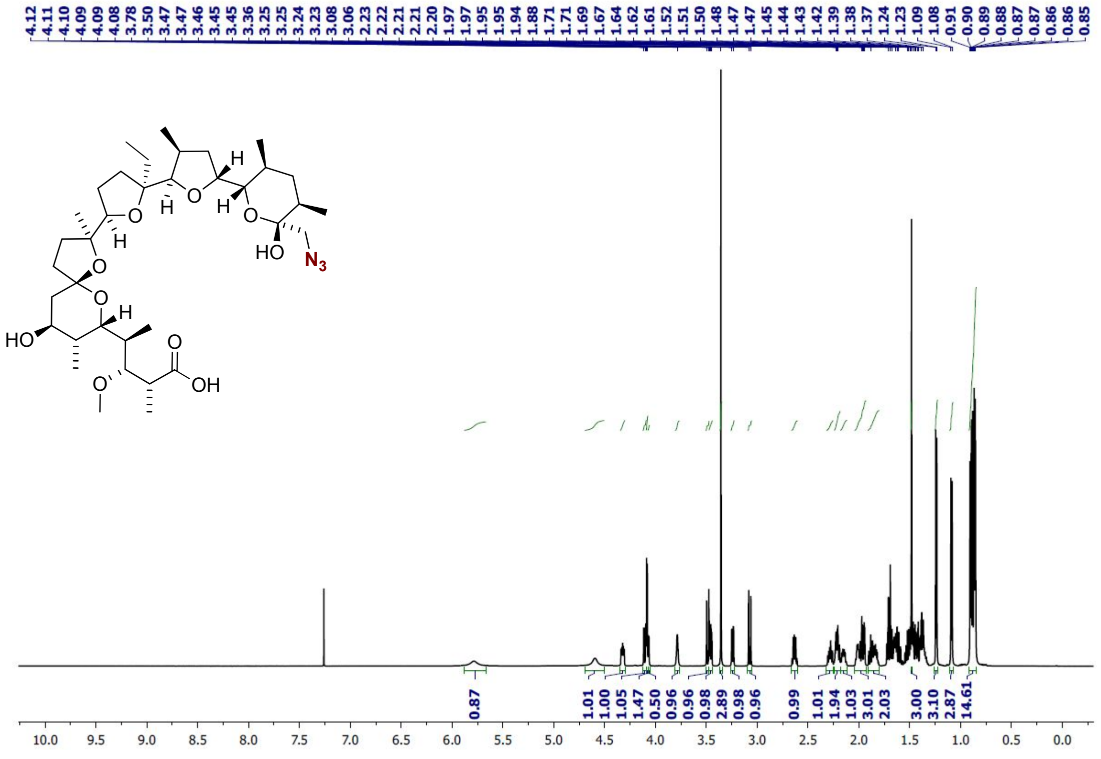

Figure S2. The ^{1}H NMR (600 MHz) spectrum of **2** in chloroform-d.

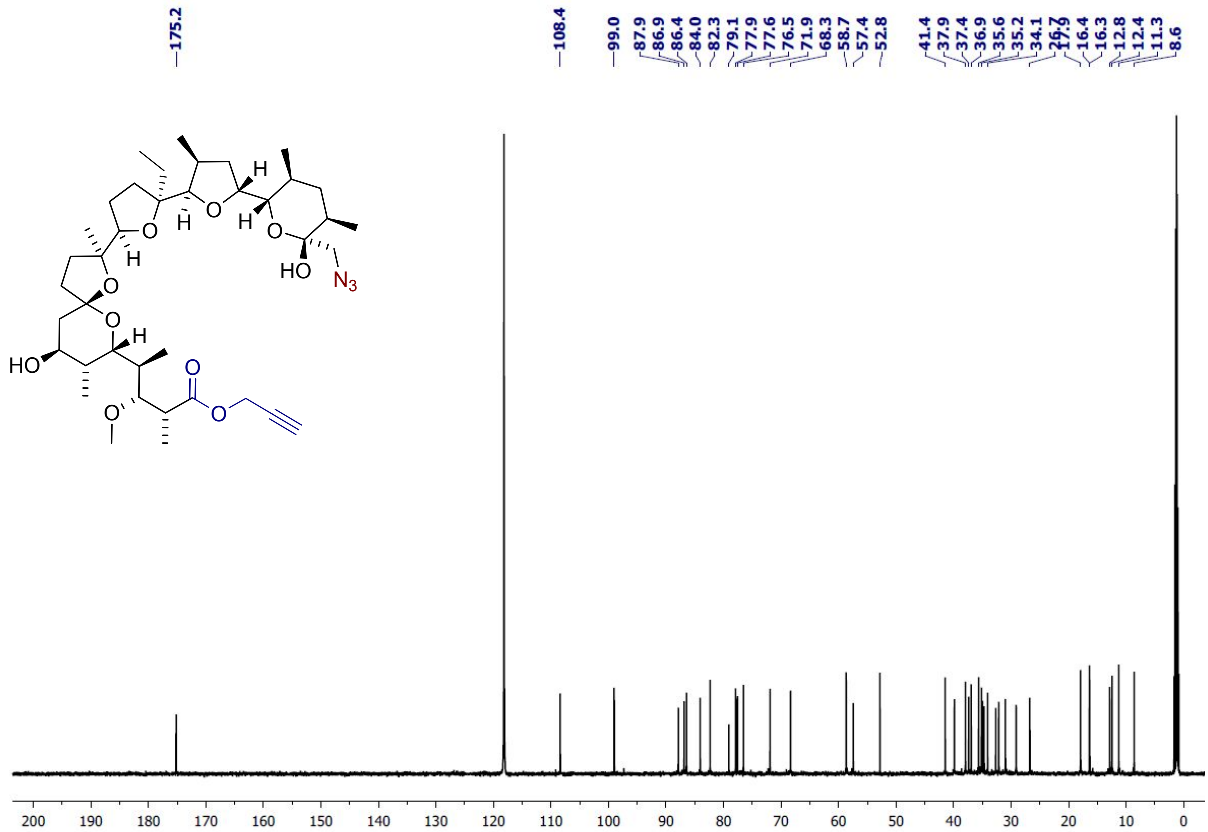

Figure S3. The $^{13}C\{^{1}H\}$ NMR (151 MHz) spectrum of **3** in acetonitrile-d3.

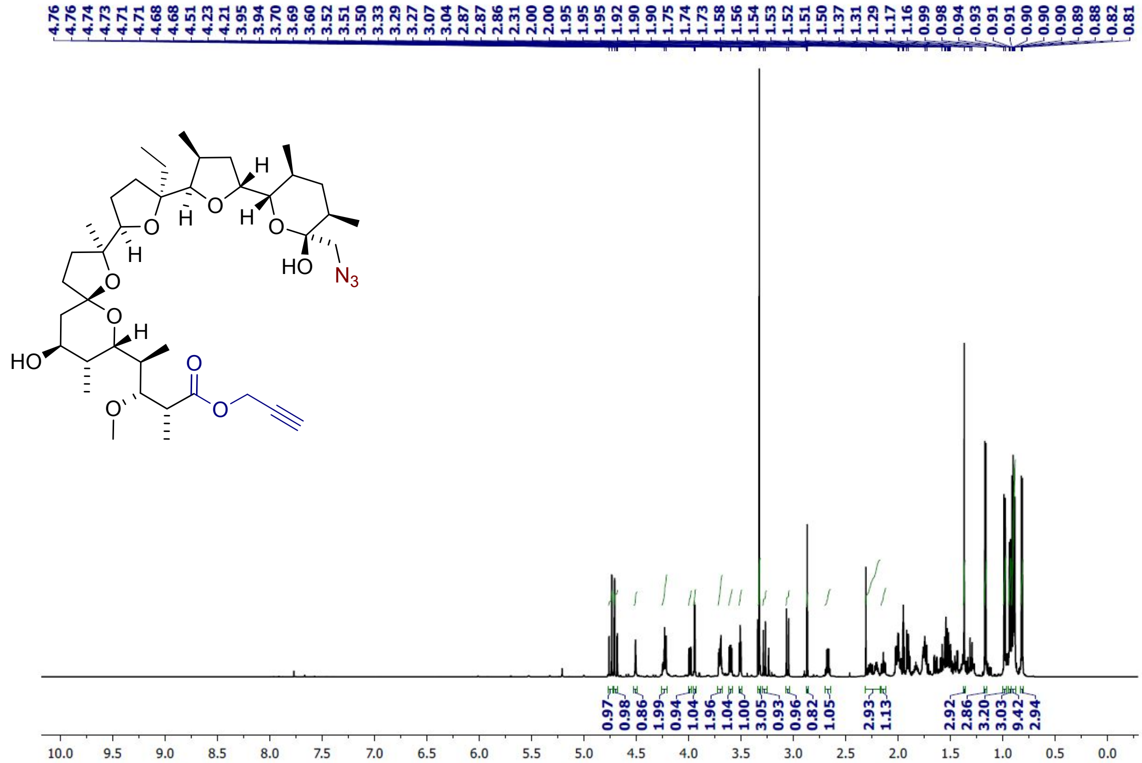

Figure S4. The ^{1}H NMR (600 MHz) spectrum of **3** in acetonitrile-d3.

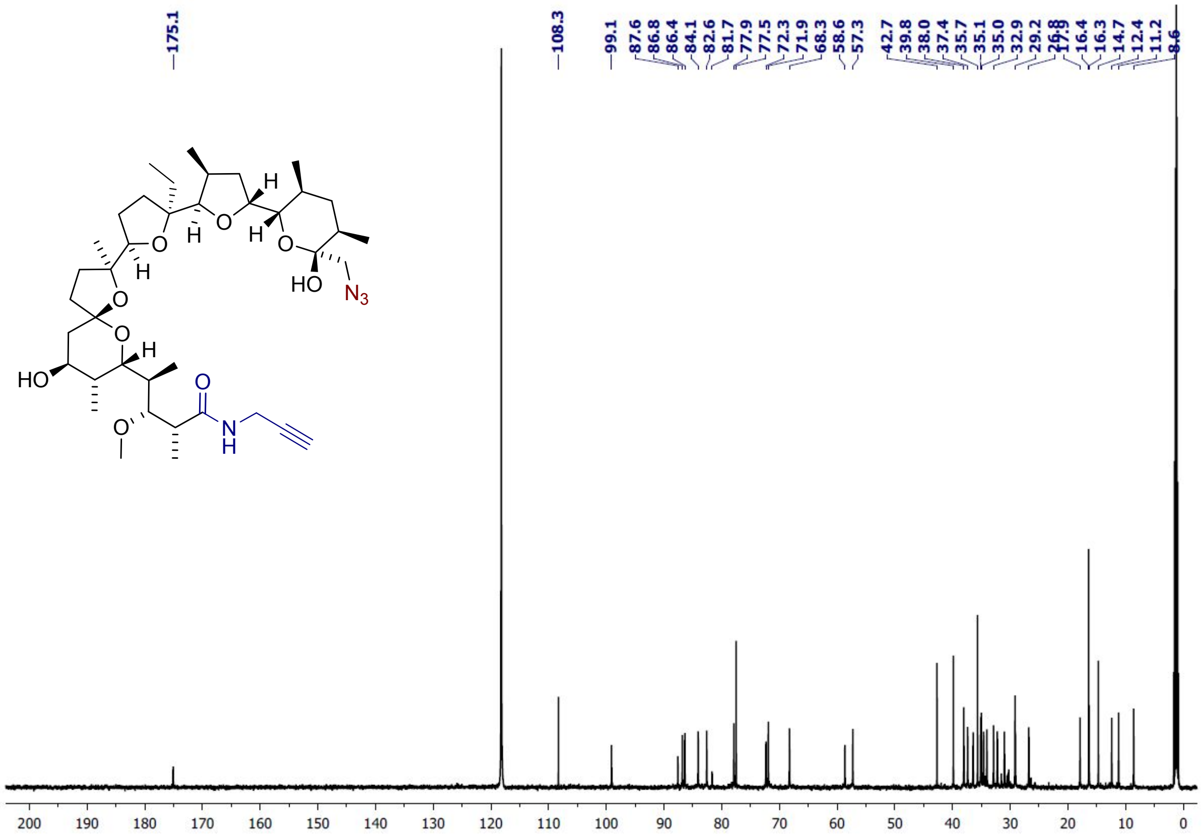

Figure S5. The $^{13}C\{^{1}H\}$ NMR (151 MHz) spectrum of **4** in acetonitrile-d3.

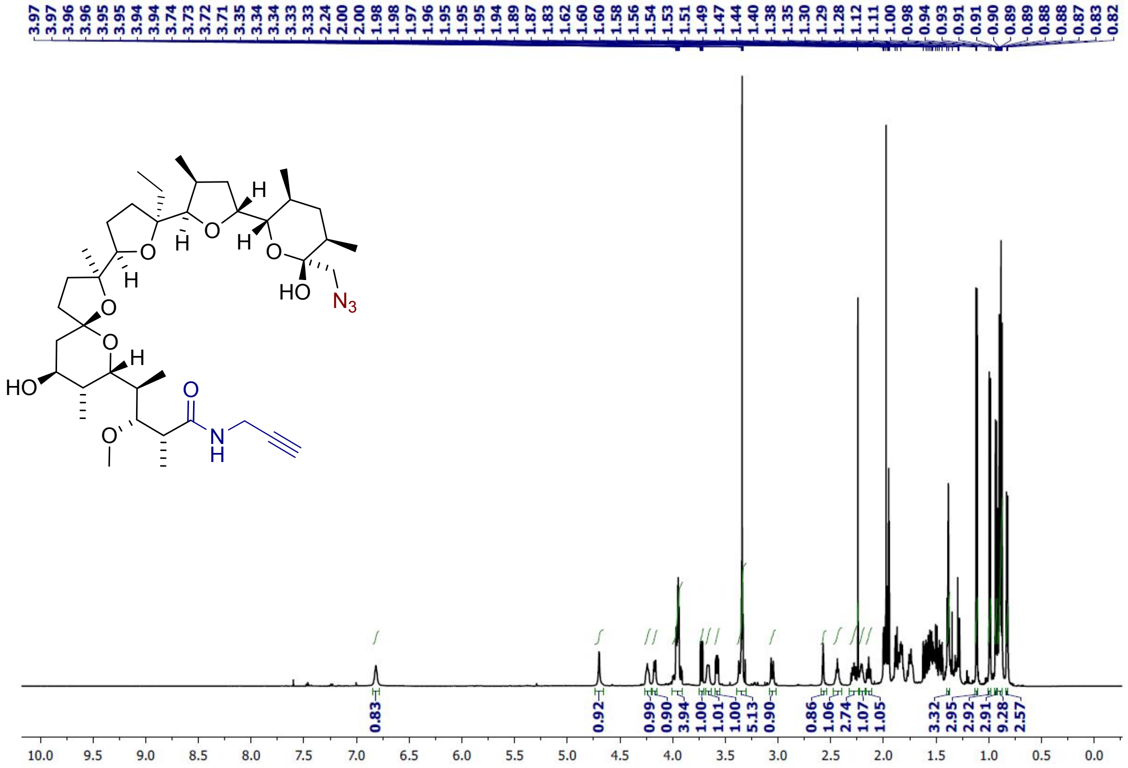

Figure S6. The ^{1}H NMR (600 MHz) spectrum of **4** in acetonitrile-d3.

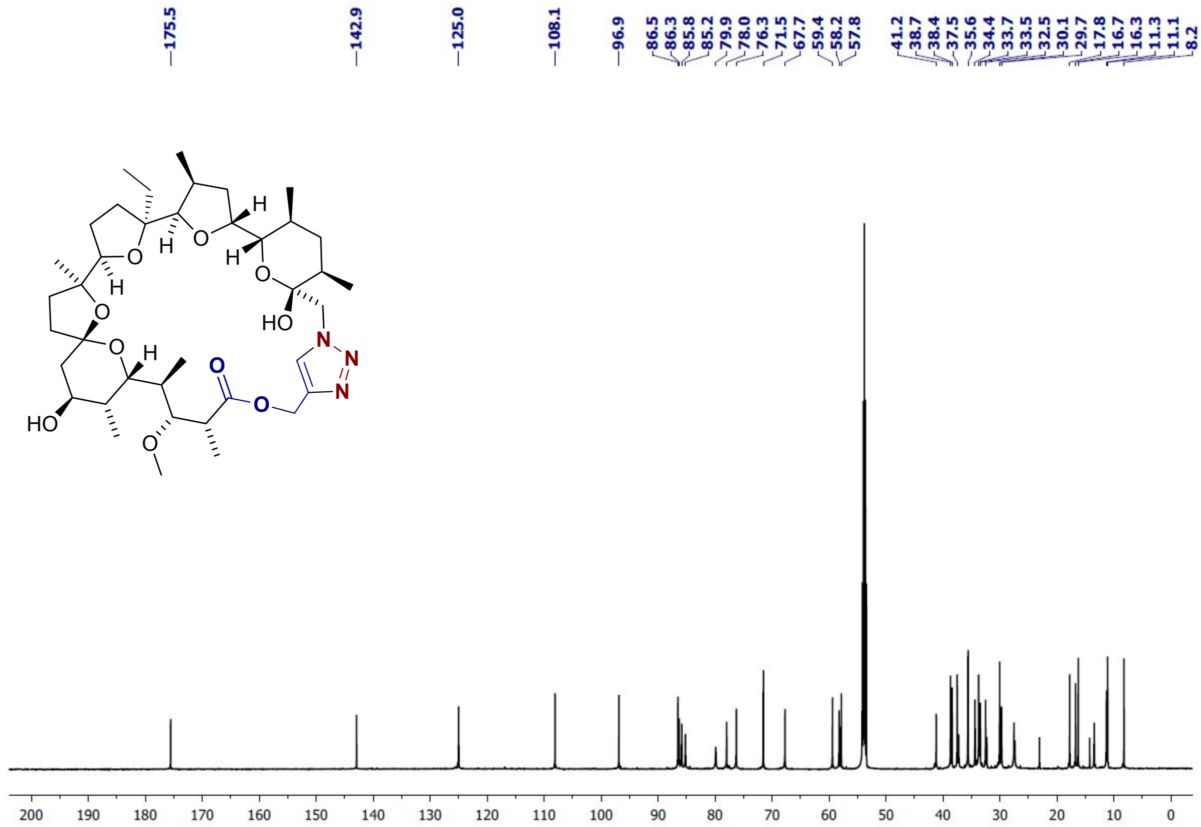

Figure S7. The $^{13}C\{^{1}H\}$ NMR (151 MHz) spectrum of **5** in dichloromethane-d2.

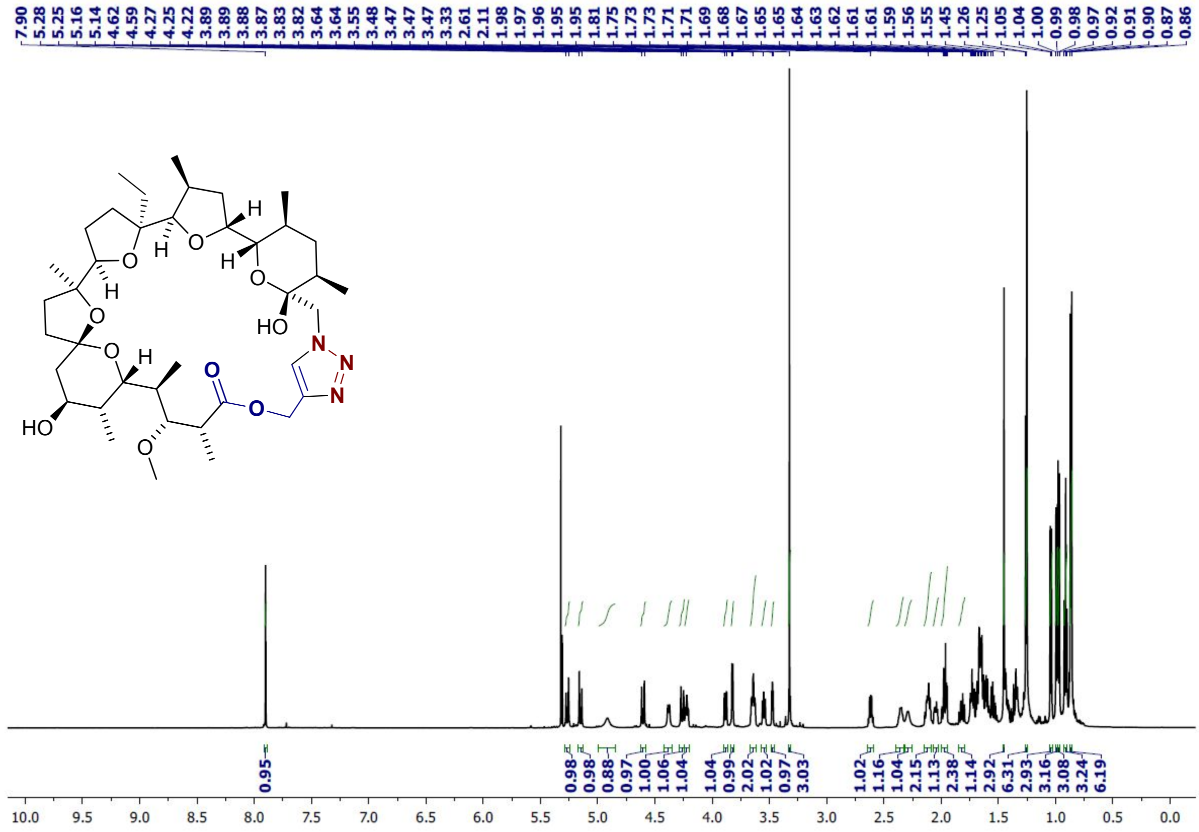

Figure S8. The ^{1}H NMR (600 MHz) spectrum of **5** in dichloromethane-d2.

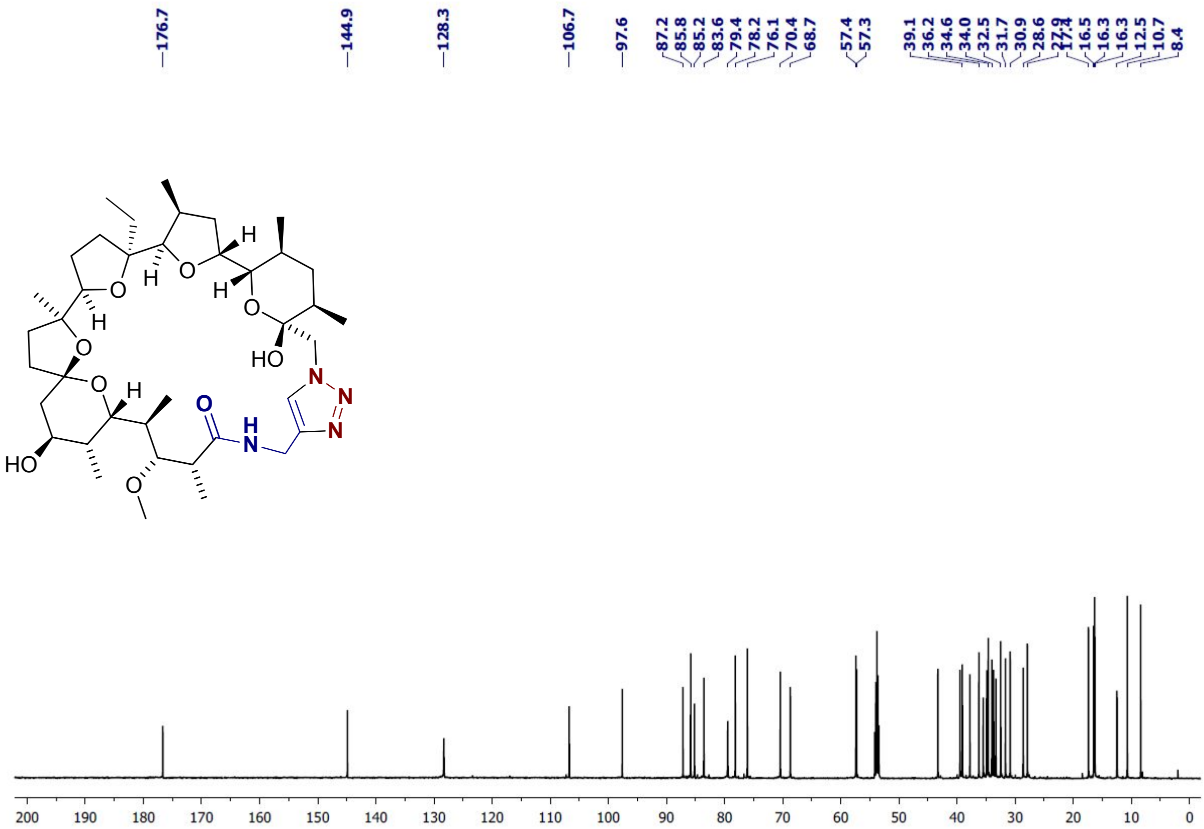

Figure S9. The $^{13}C\{^1H\}$ NMR (151 MHz) spectrum of **6** in dichloromethane-d2.

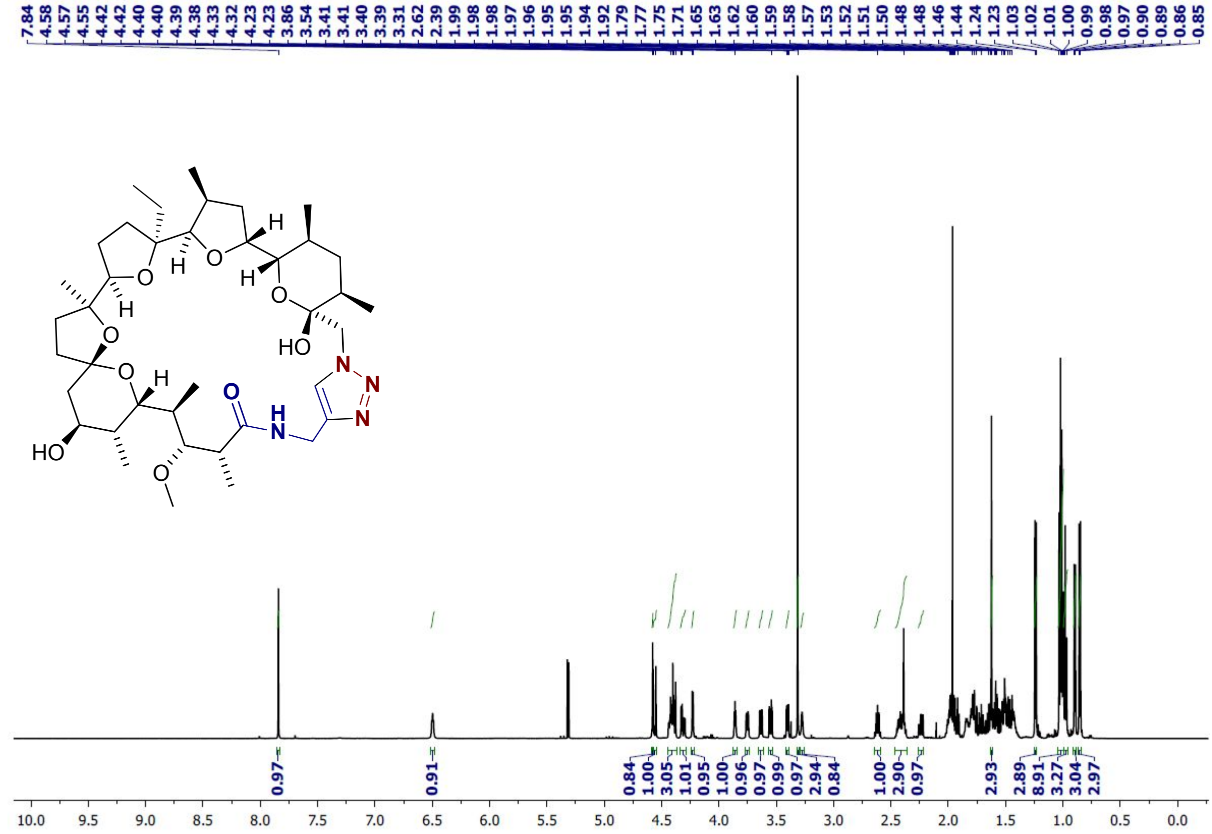

Figure S10. The ^1H NMR (600 MHz) spectrum of **6** in dichloromethane-d2.

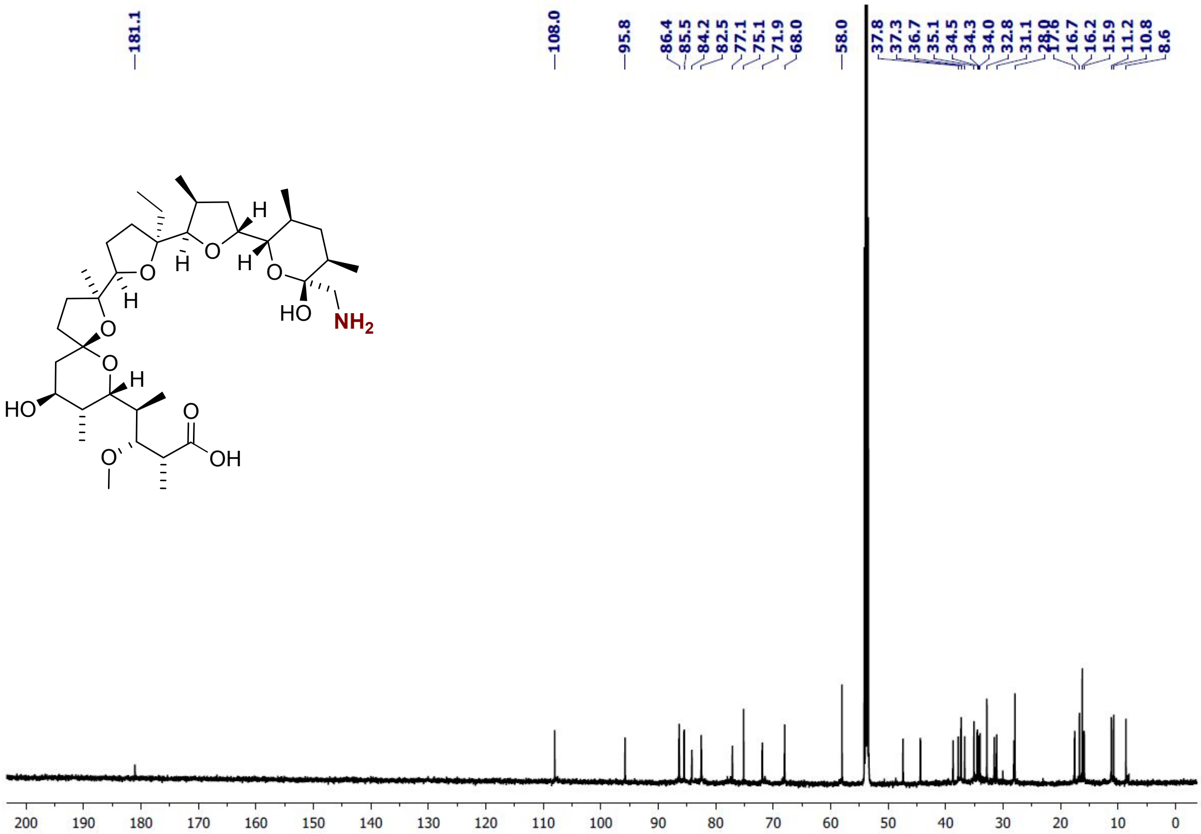

Figure S11. The $^{13}C\{^{1}H\}$ NMR (151 MHz) spectrum of **7** in dichloromethane-d2.

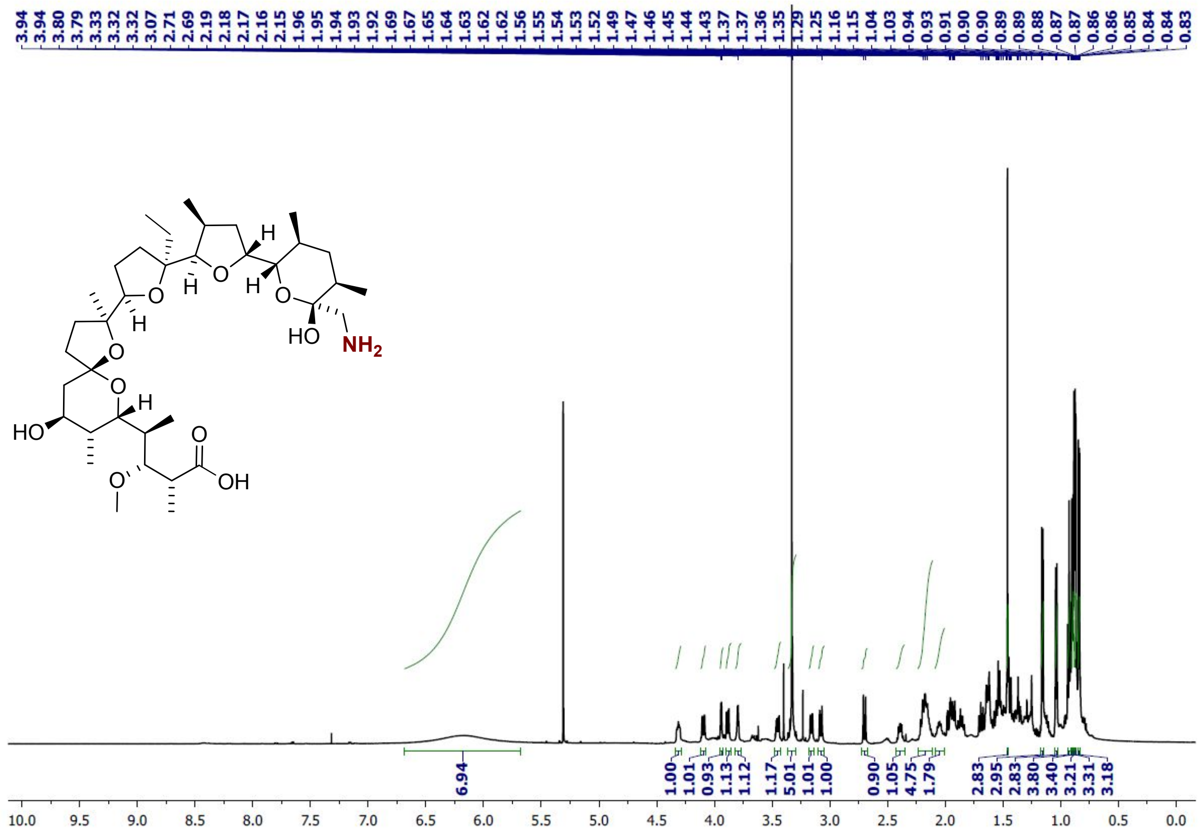

Figure S12. The ^{1}H NMR (600 MHz) spectrum of **7** in dichloromethane-d2.

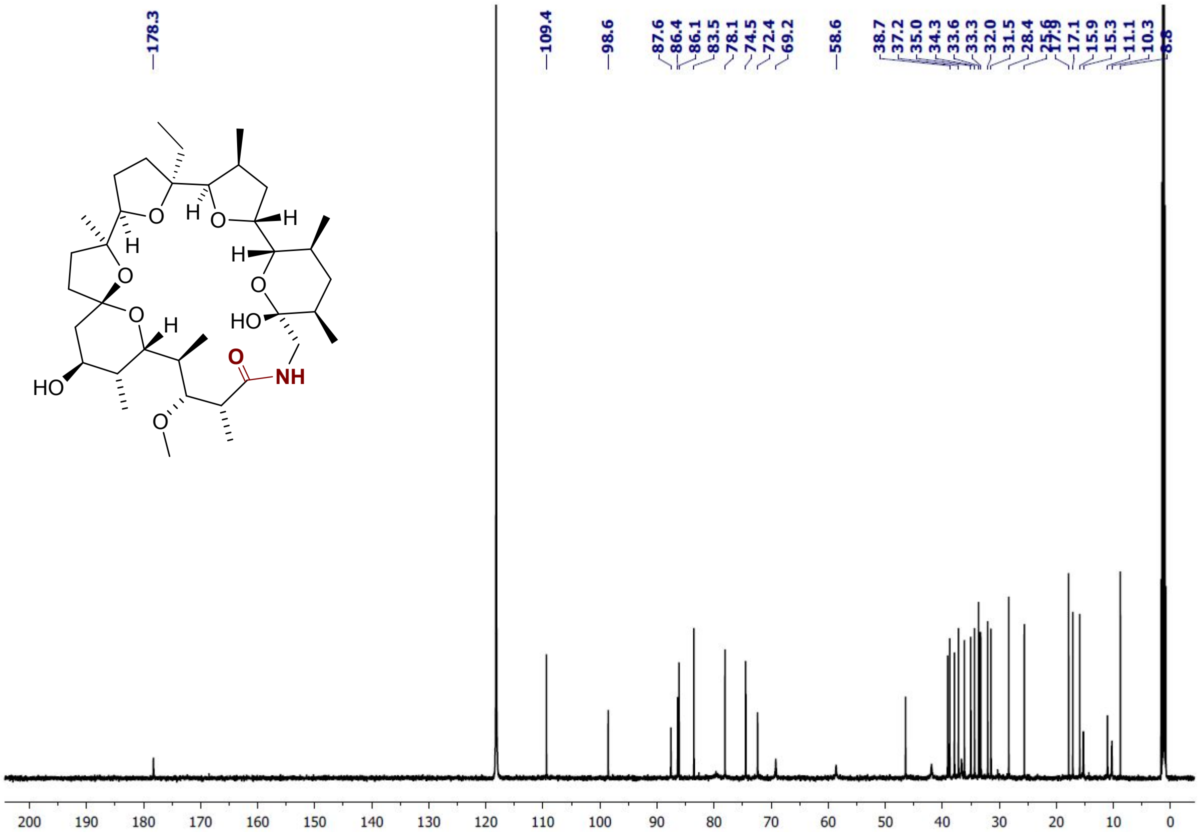

Figure S13. The $^{13}C\{^{1}H\}$ NMR (151 MHz) spectrum of **8** in acetonitrile-d3.

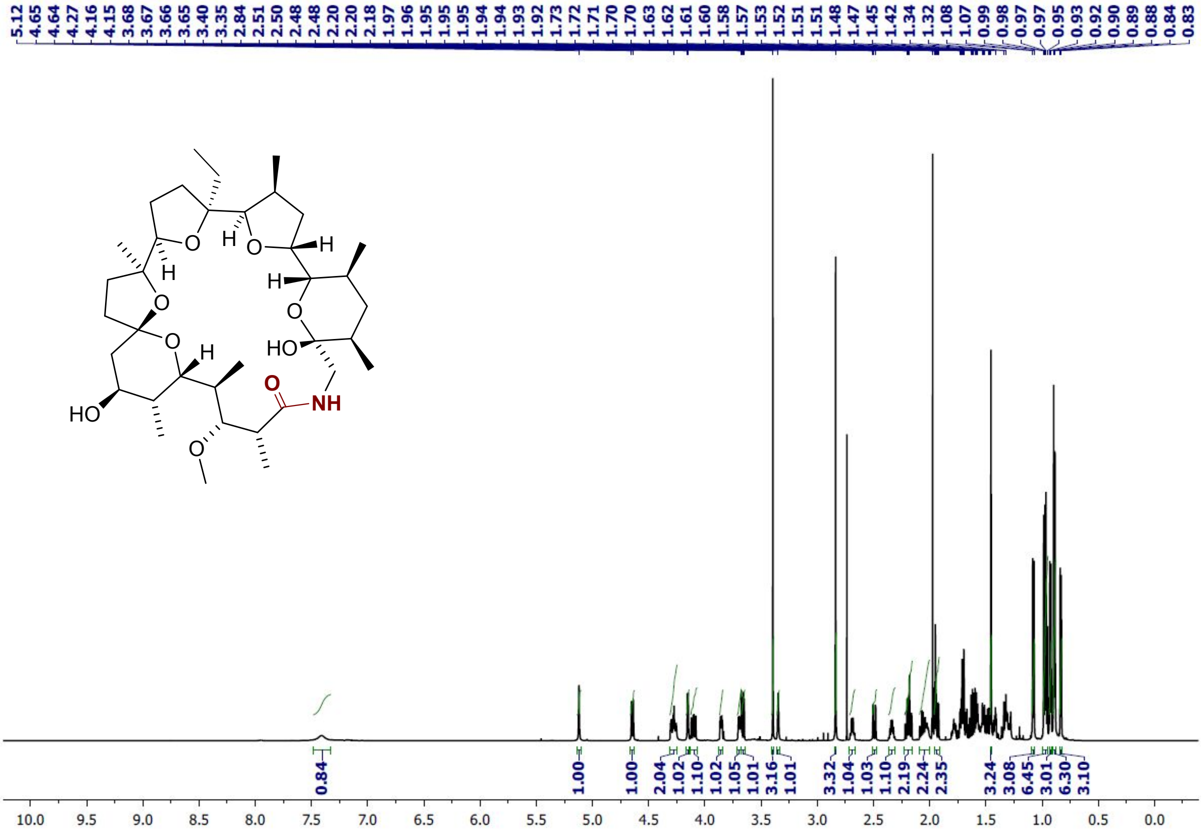

Figure S14. The ^{1}H NMR (600 MHz) spectrum of **8** in acetonitrile-d3.

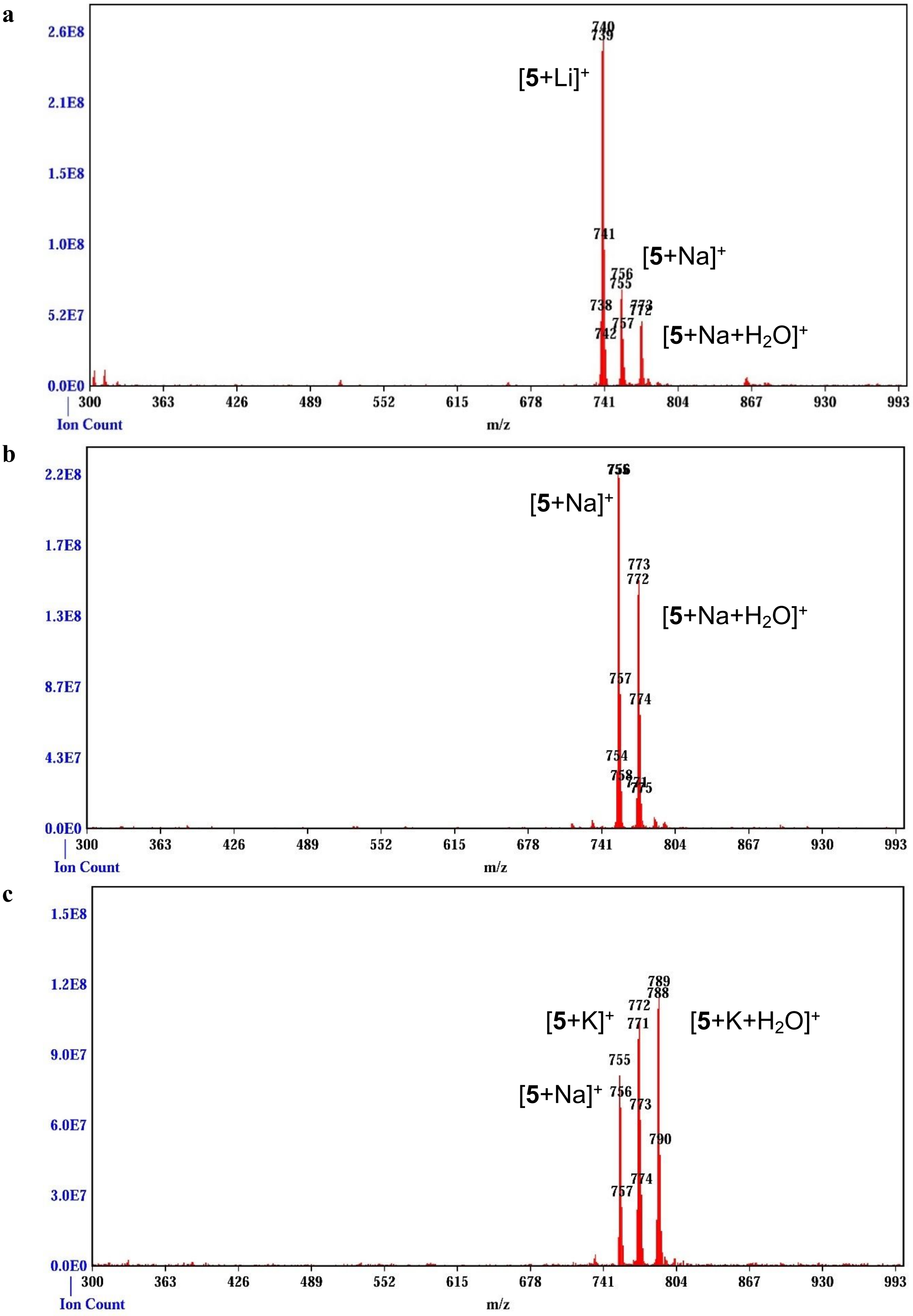

Figure S15. The ESI mass spectra of a mixture of **5** with (**a**) $LiClO_4$, (**b**) $NaClO_4$, and (**c**) $KClO_4$.

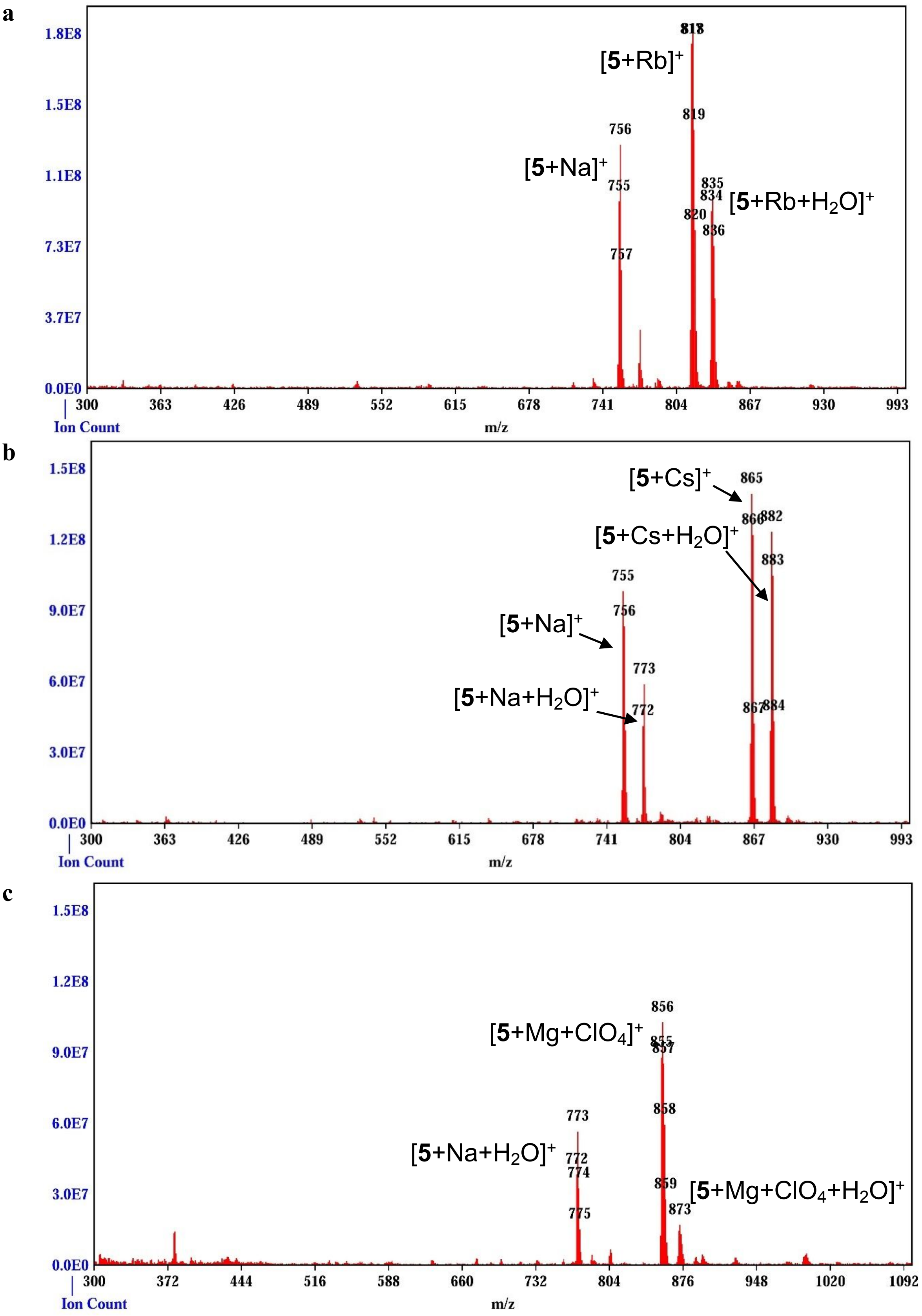

Figure S16. The ESI mass spectra of a mixture of **5** with (**a**) $RbClO_4$, (**b**) $CsClO_4$, and (**c**) $Mg(ClO_4)_2$.

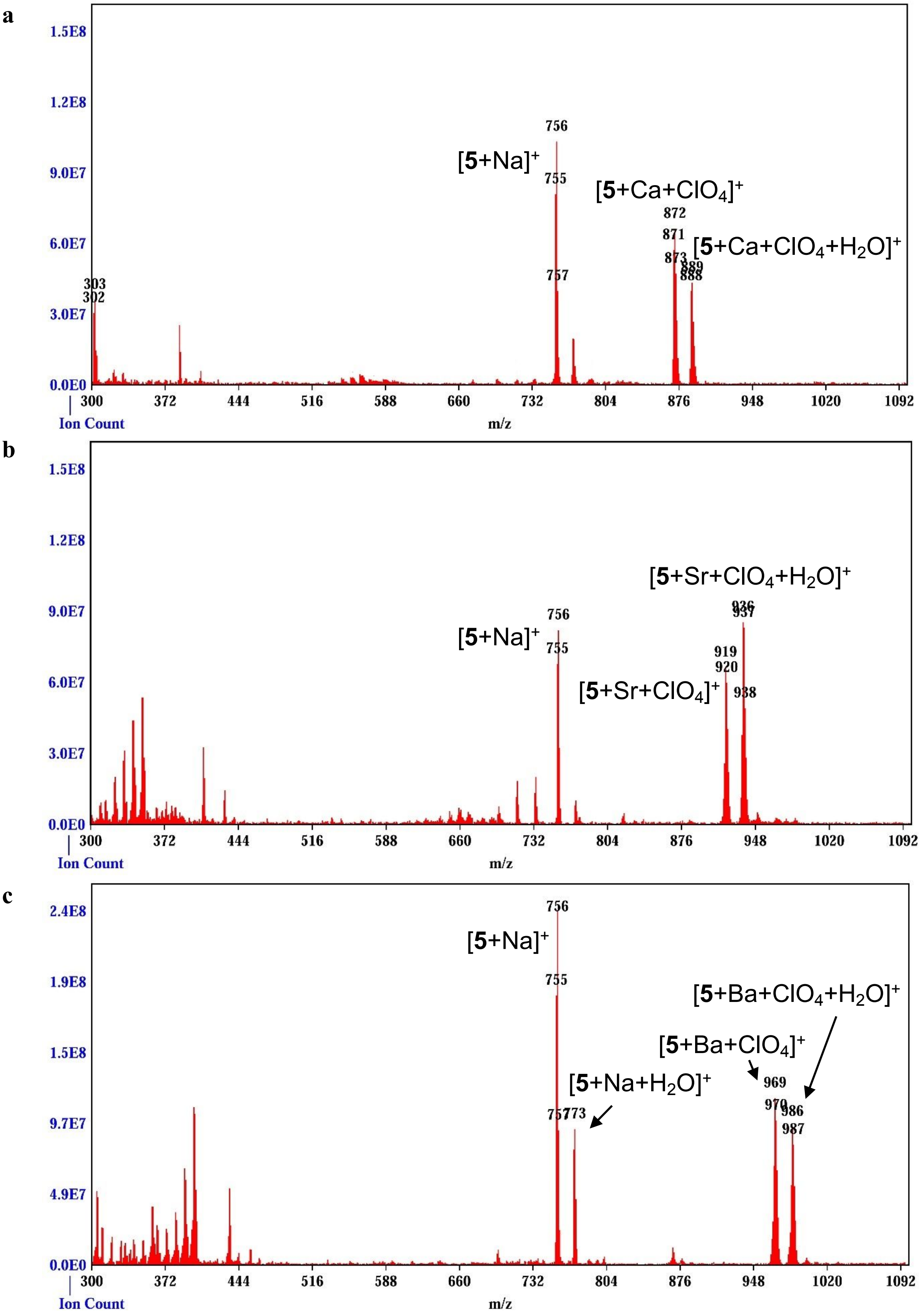

Figure S17. The ESI mass spectra of a mixture of **5** with (**a**) $Ca(ClO_4)_2$, (**b**) $Sr(ClO_4)_2$, and (**c**) $Ba(ClO_4)_2$.

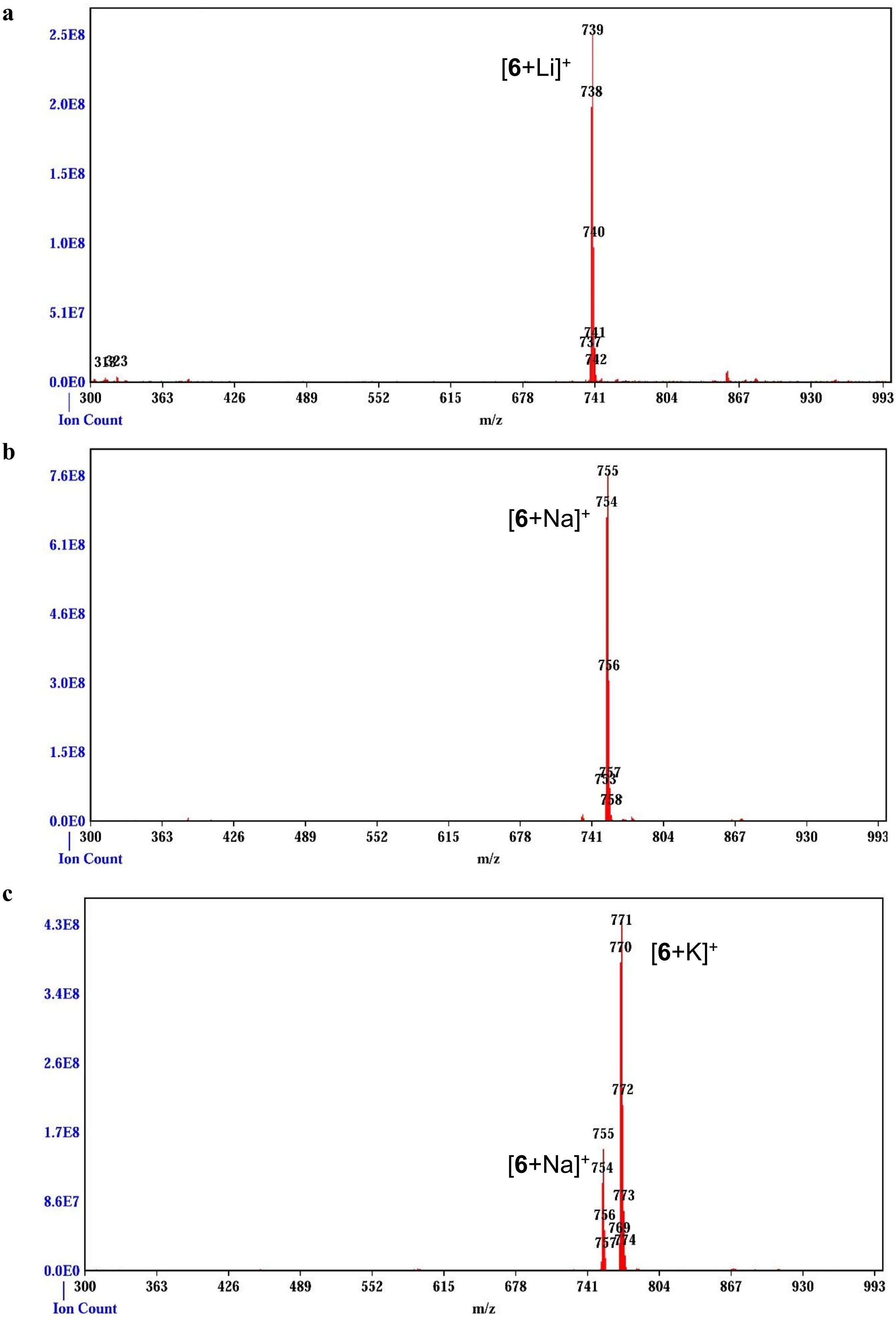

Figure S18. The ESI mass spectra of a mixture of **6** with (**a**) $LiClO_4$, (**b**) $NaClO_4$, and (**c**) $KClO_4$.

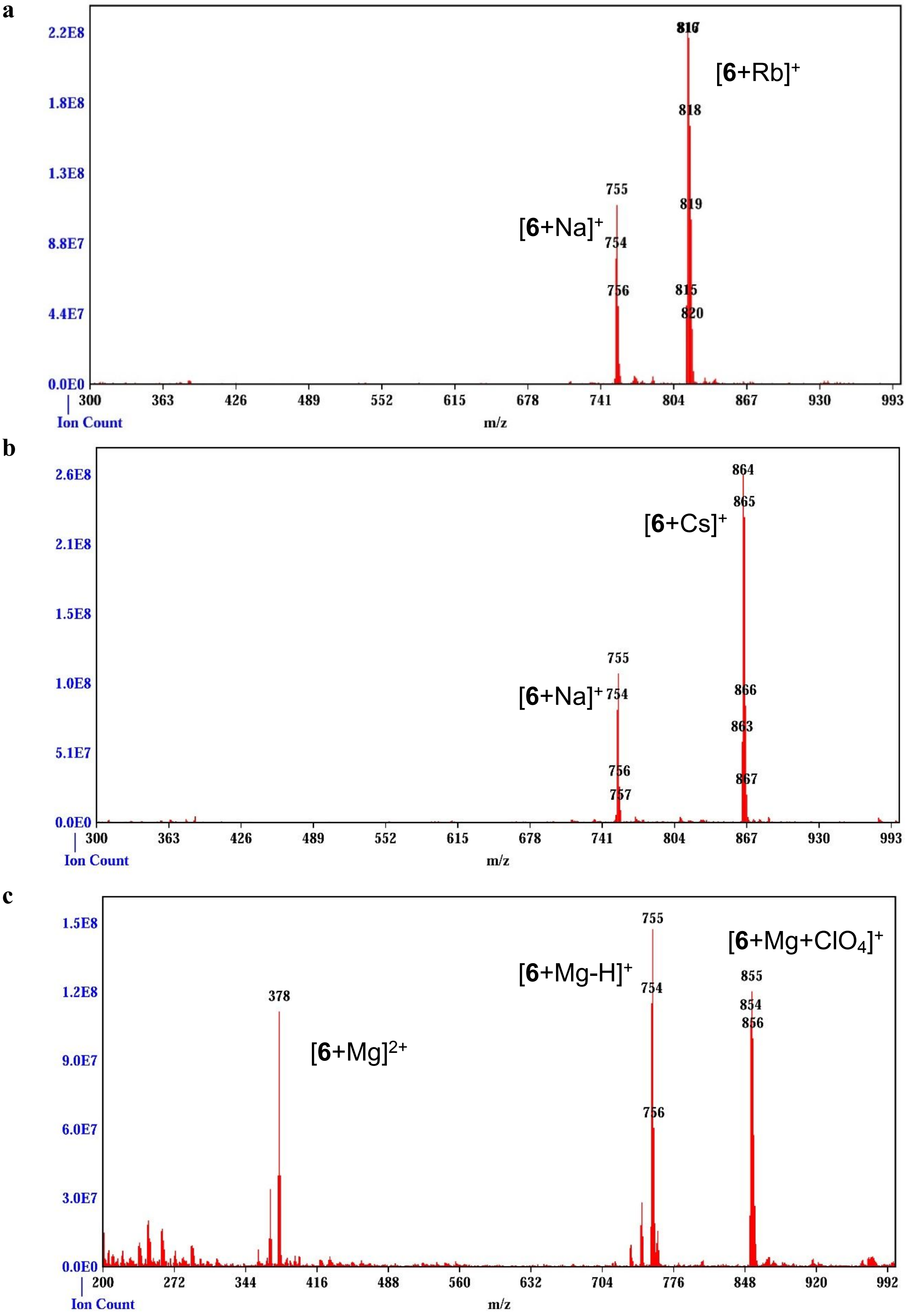

Figure S19. The ESI mass spectra of a mixture of **6** with (**a**) $RbClO_4$, (**b**) $CsClO_4$, and (**c**) $Mg(ClO_4)_2$.

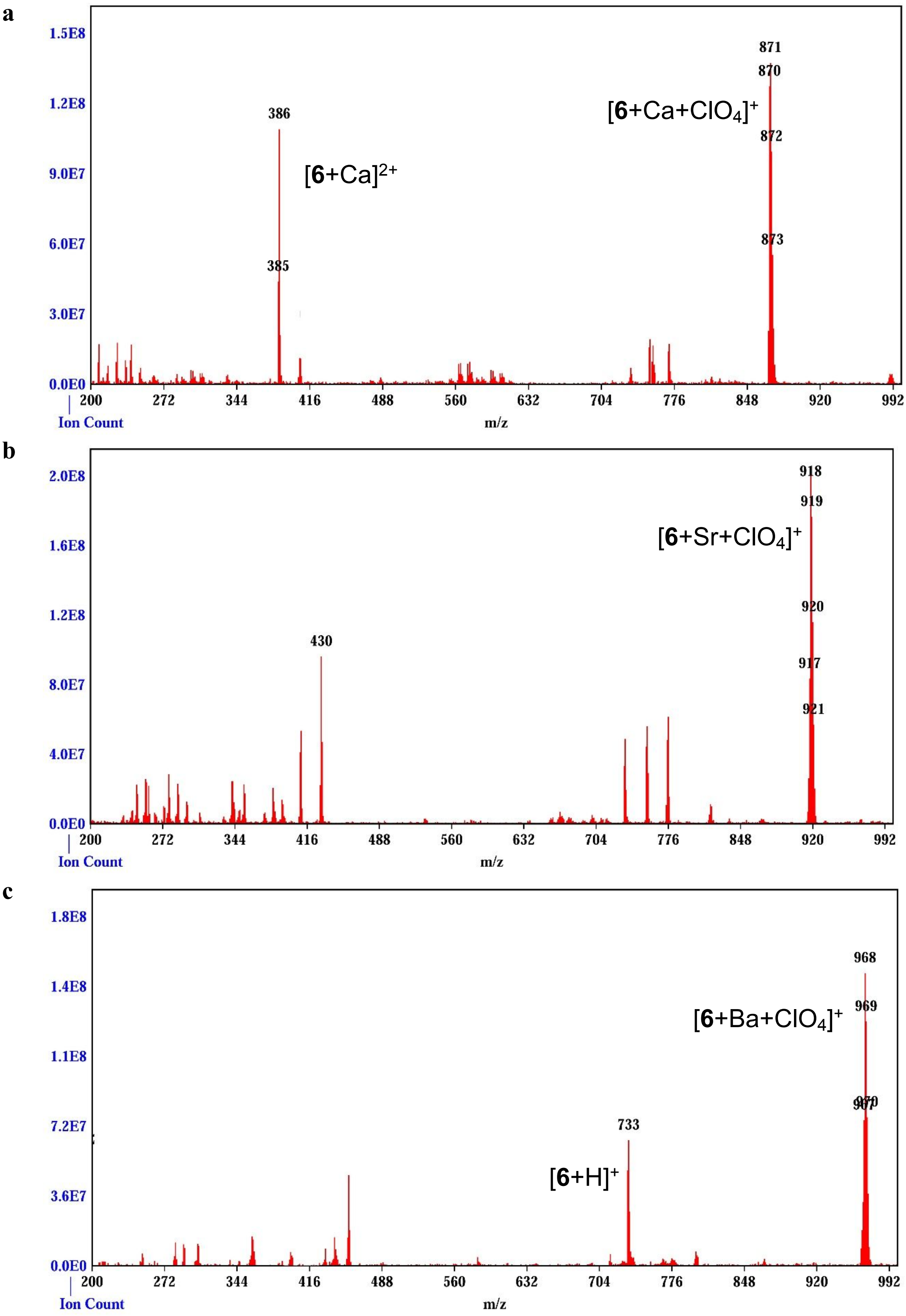

Figure S20. The ESI mass spectra of a mixture of **6** with (**a**) $Ca(ClO_4)_2$, (**b**) $Sr(ClO_4)_2$, and (**c**) $Ba(ClO_4)_2$.

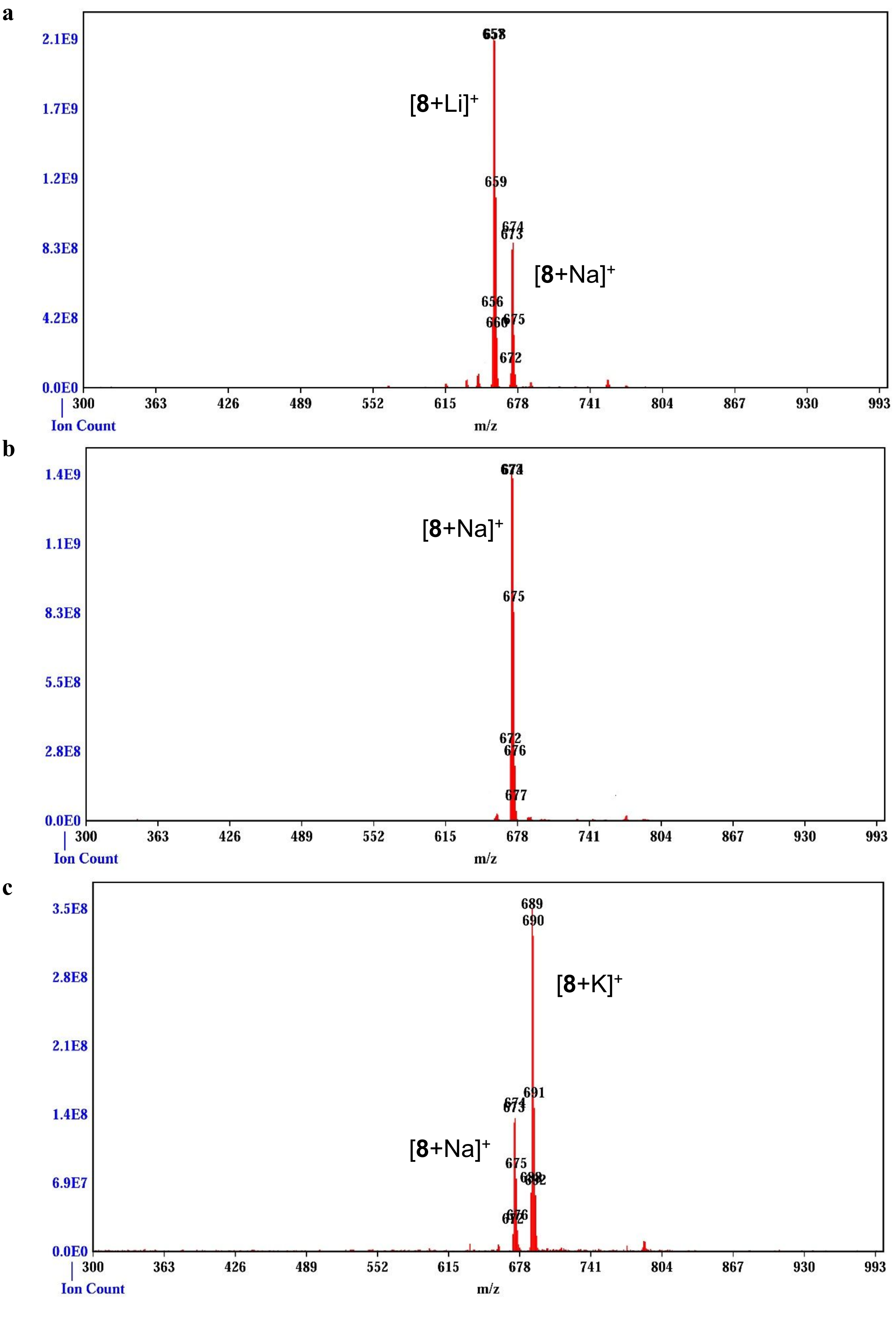

Figure S21. The ESI mass spectra of a mixture of **8** with (**a**) $LiClO_4$, (**b**) $NaClO_4$, and (**c**) $KClO_4$.

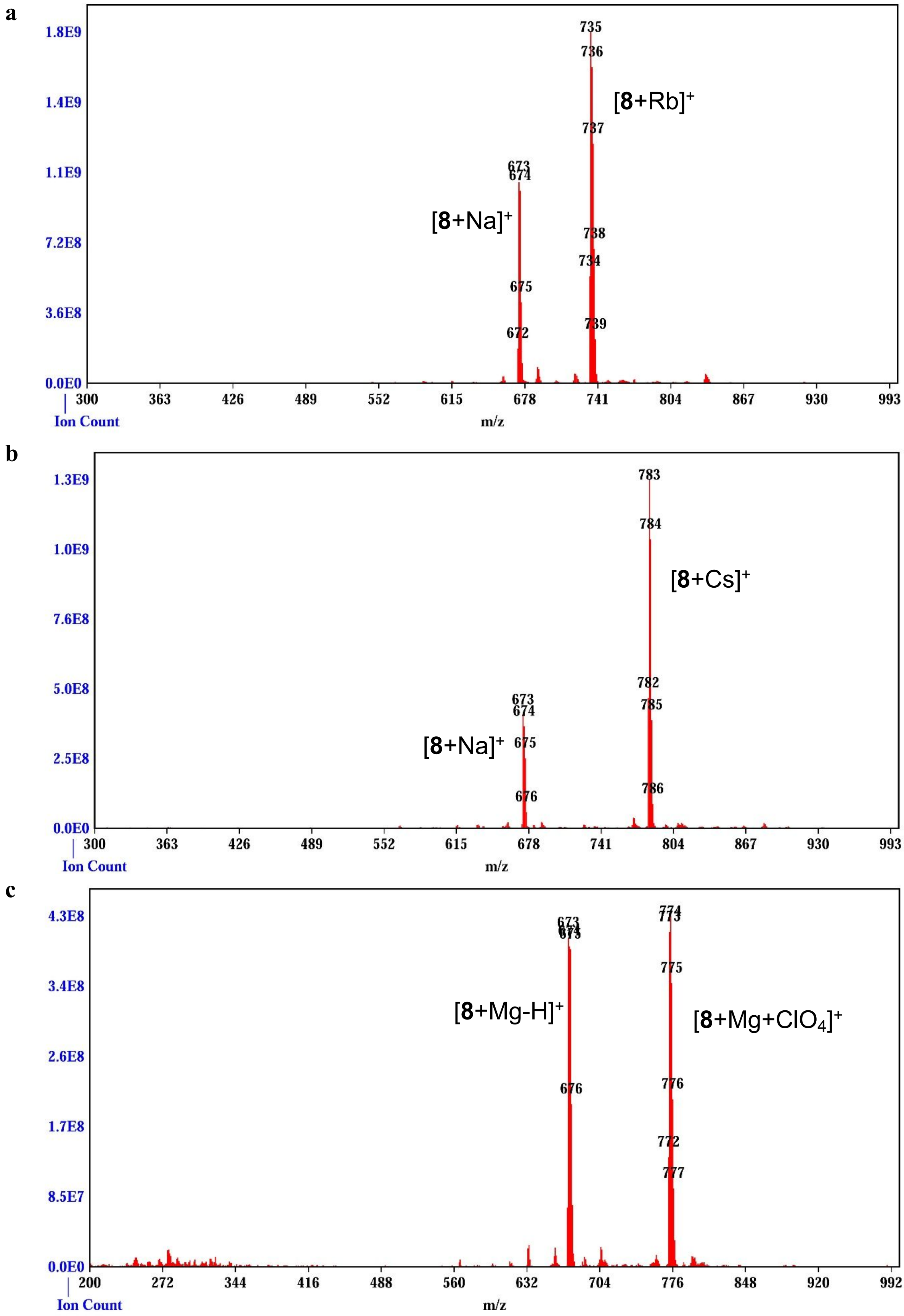

Figure S22. The ESI mass spectra of a mixture of **8** with (**a**) $RbClO_4$, (**b**) $CsClO_4$, and (**c**) $Mg(ClO_4)_2$.

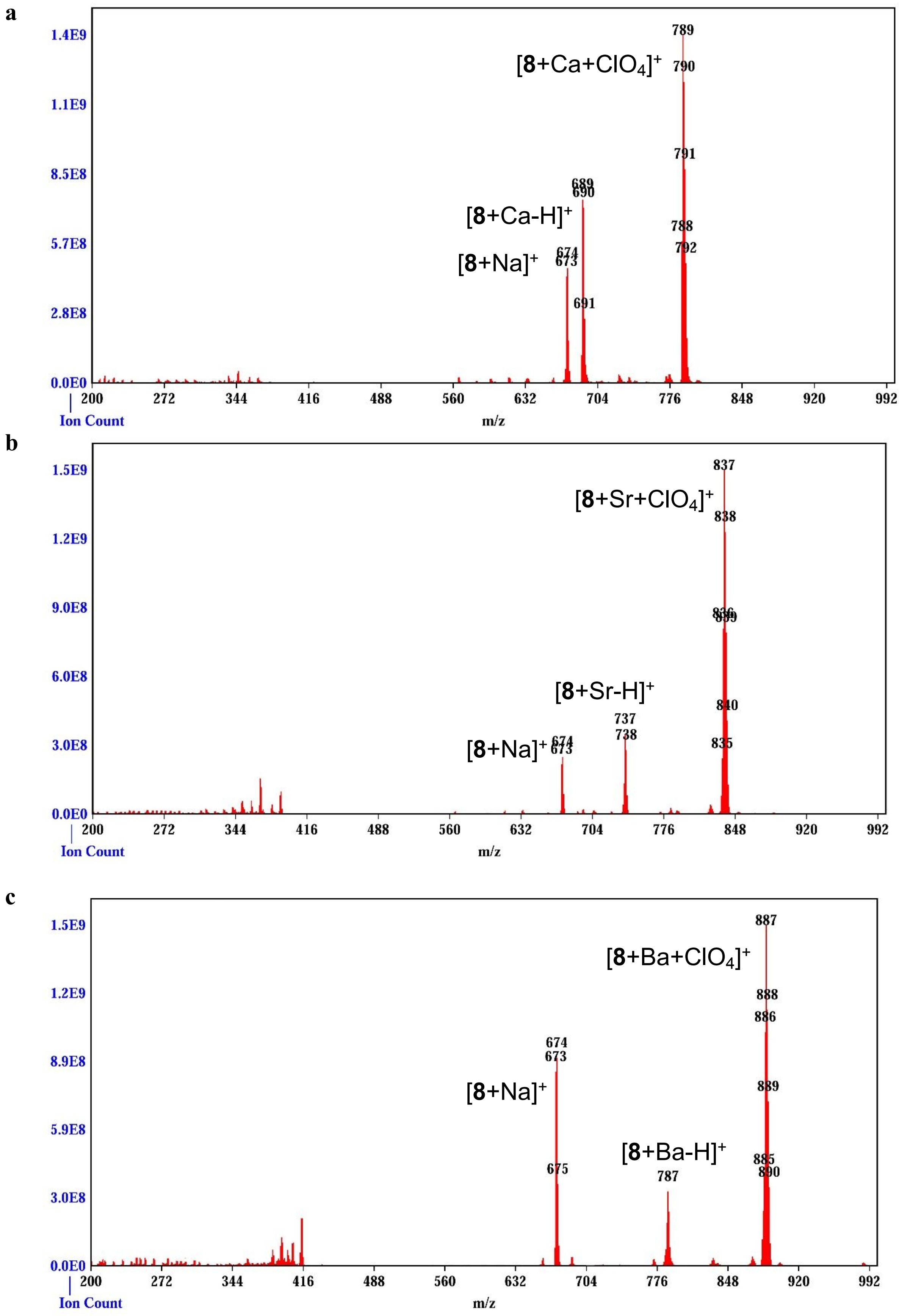

Figure S23. The ESI mass spectra of a mixture of **8** with (**a**) $Ca(ClO_4)_2$, (**b**) $Sr(ClO_4)_2$, and (**c**) $Ba(ClO_4)_2$.

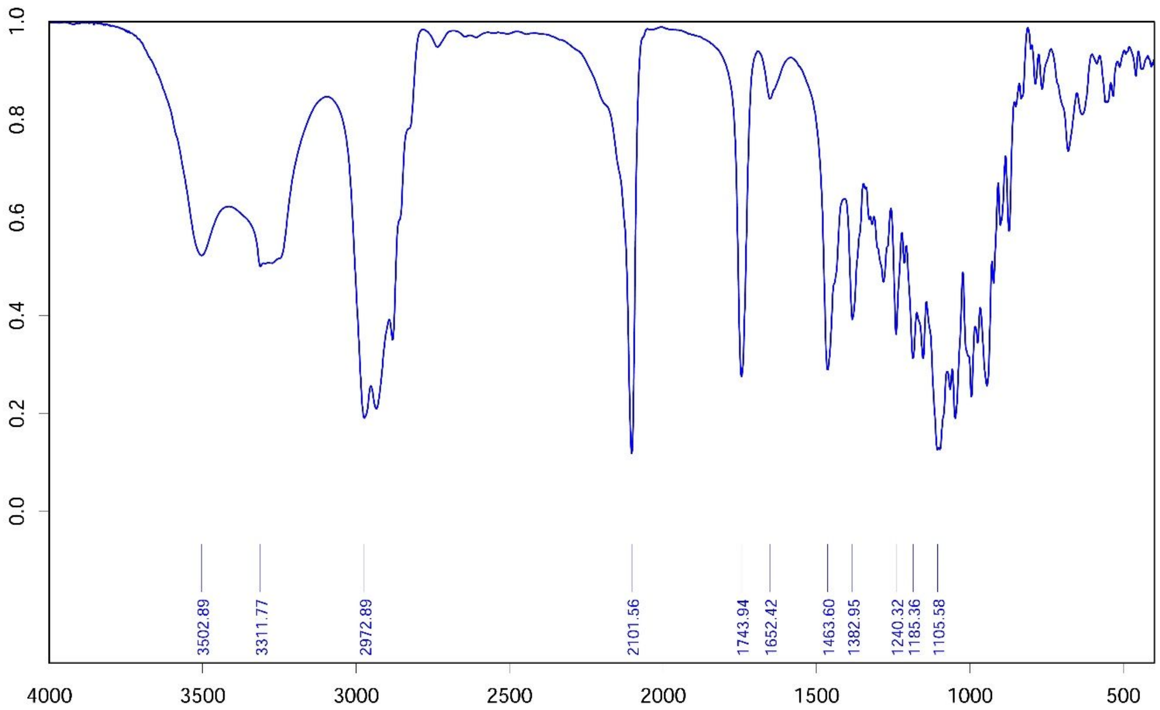

Figure S24. The FT-IR spectrum of **3** in the range of 4000-500 cm^{-1}.

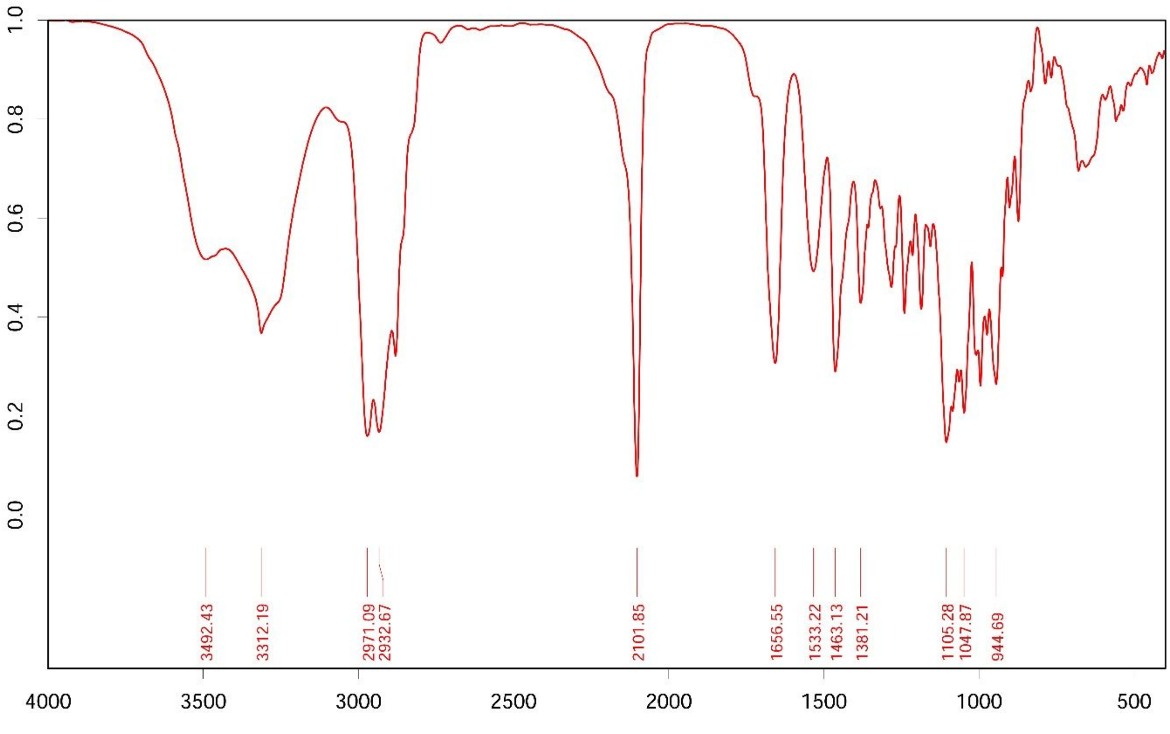

Figure S25. The FT-IR spectrum of **4** in the range of 4000-500 cm^{-1}.

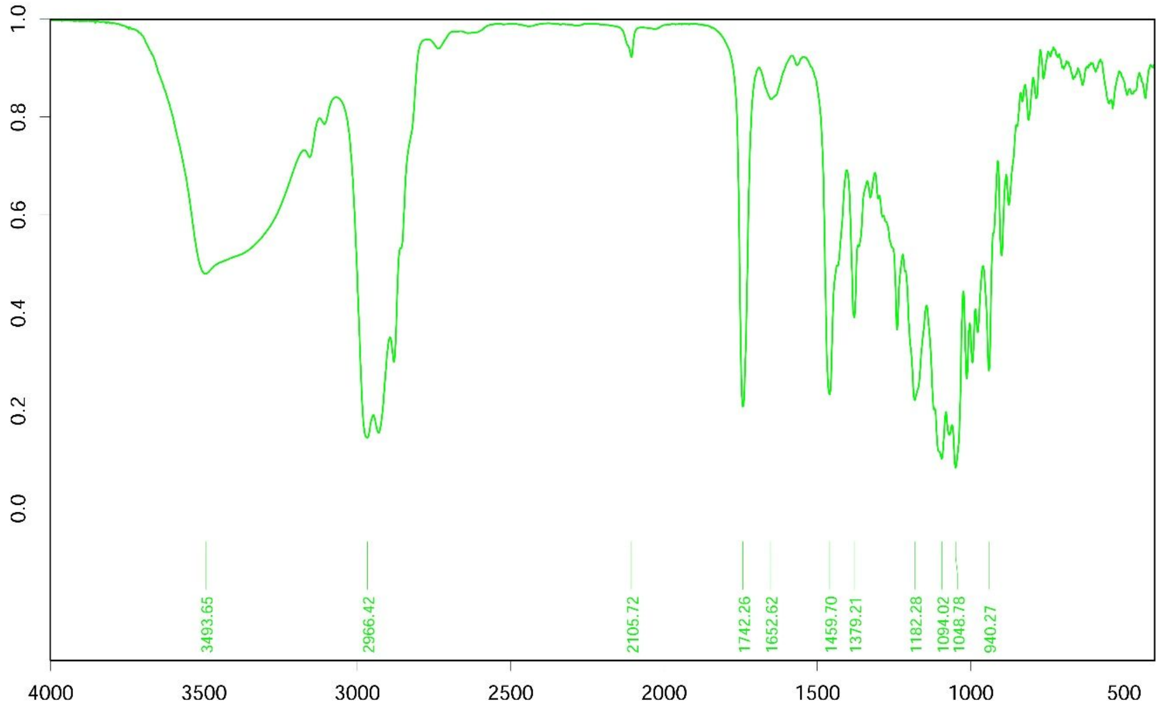

Figure S26. The FT-IR spectrum of **5** in the range of 4000-500 cm^{-1}.

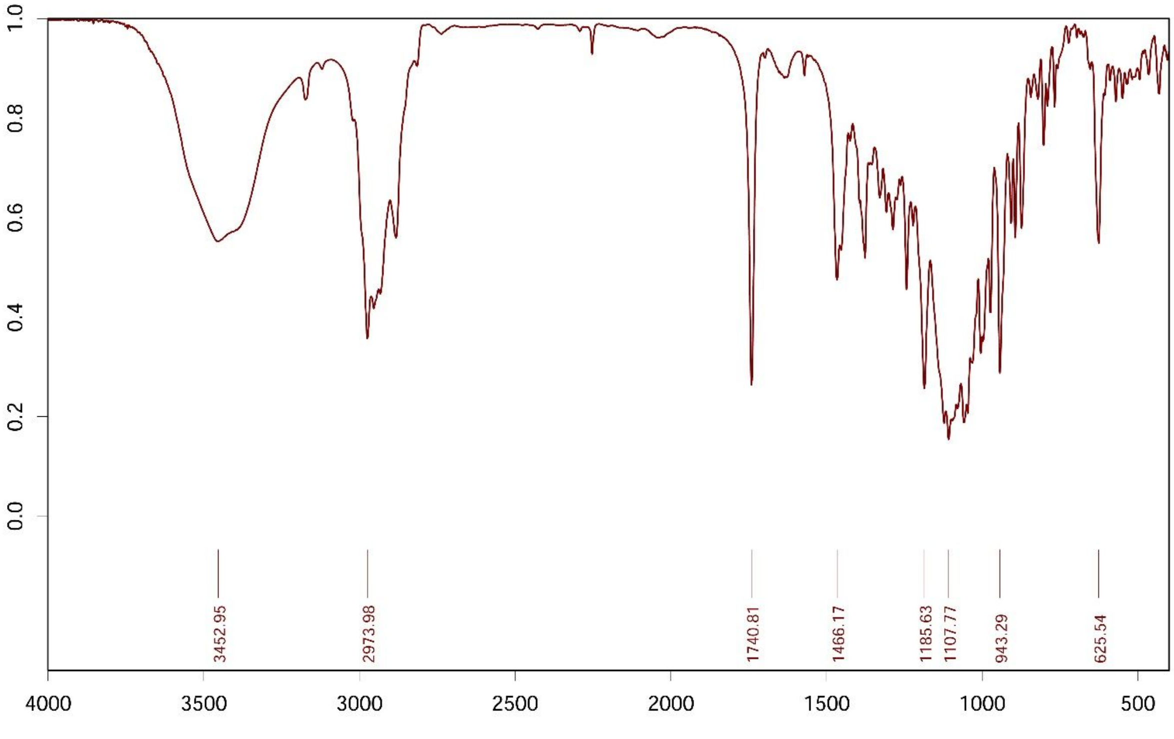

Figure S27. The FT-IR spectrum of **5** with the addition of $NaClO_4$ in the range of 4000-500 cm^{-1}.

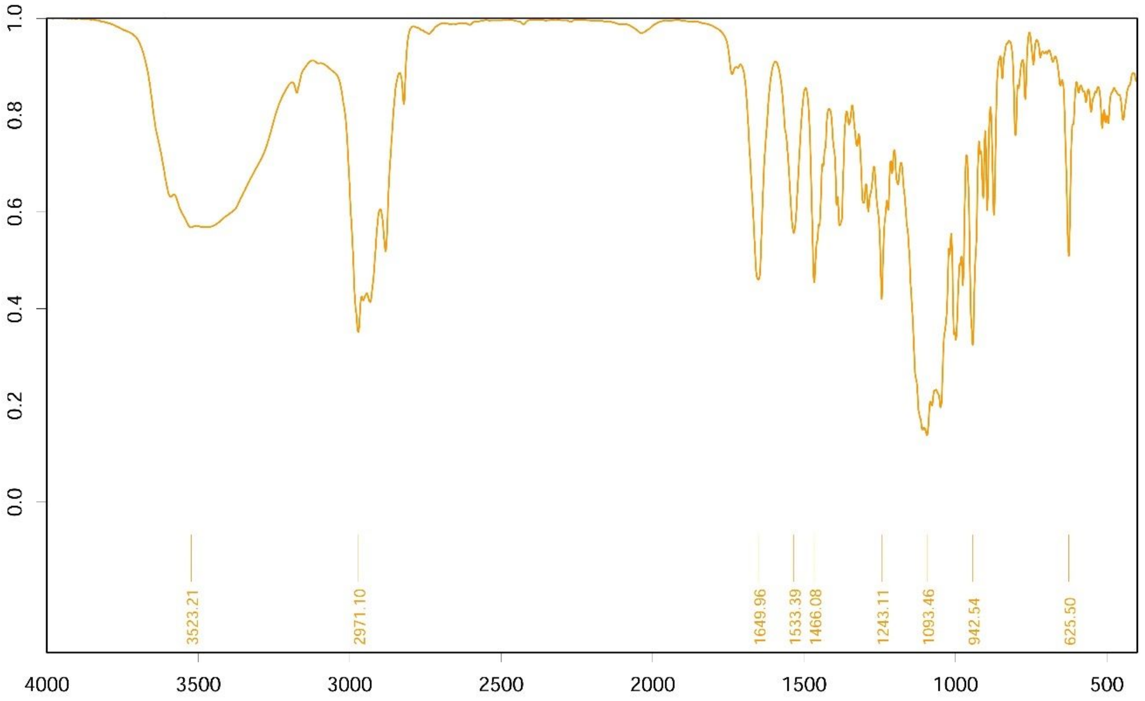

Figure S28. The FT-IR spectrum of **6** in the range of 4000-500 cm^{-1}.

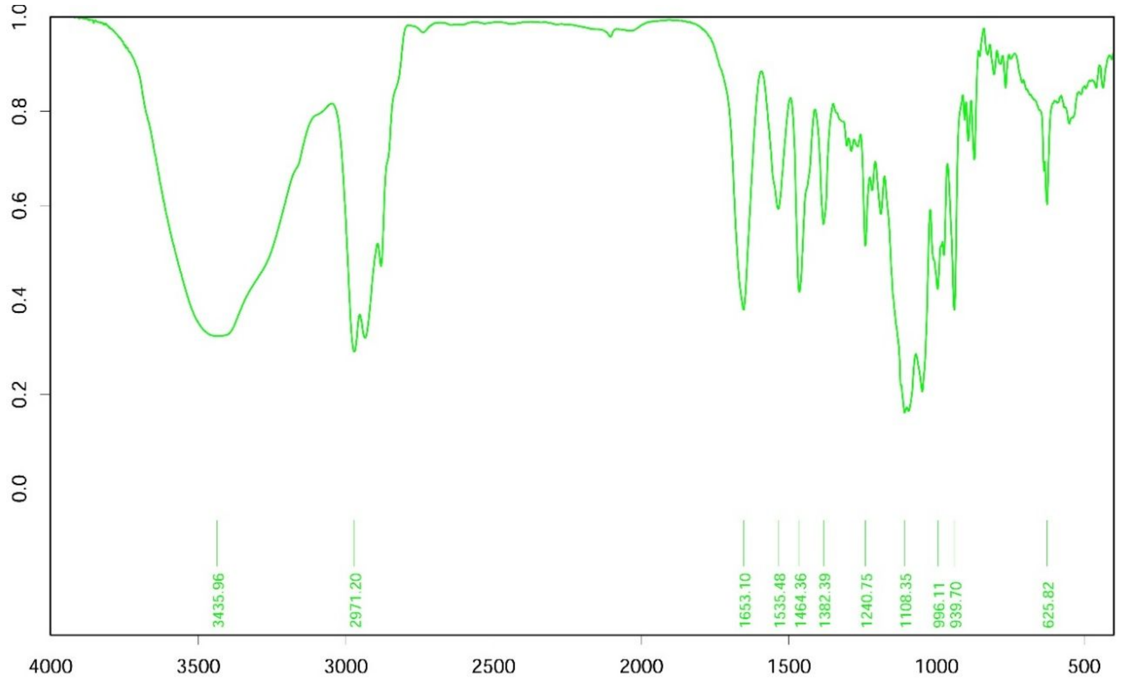

Figure S29. The FT-IR spectrum of **6** with the addition of $NaClO_4$ in the range of 4000-500 cm^{-1}.

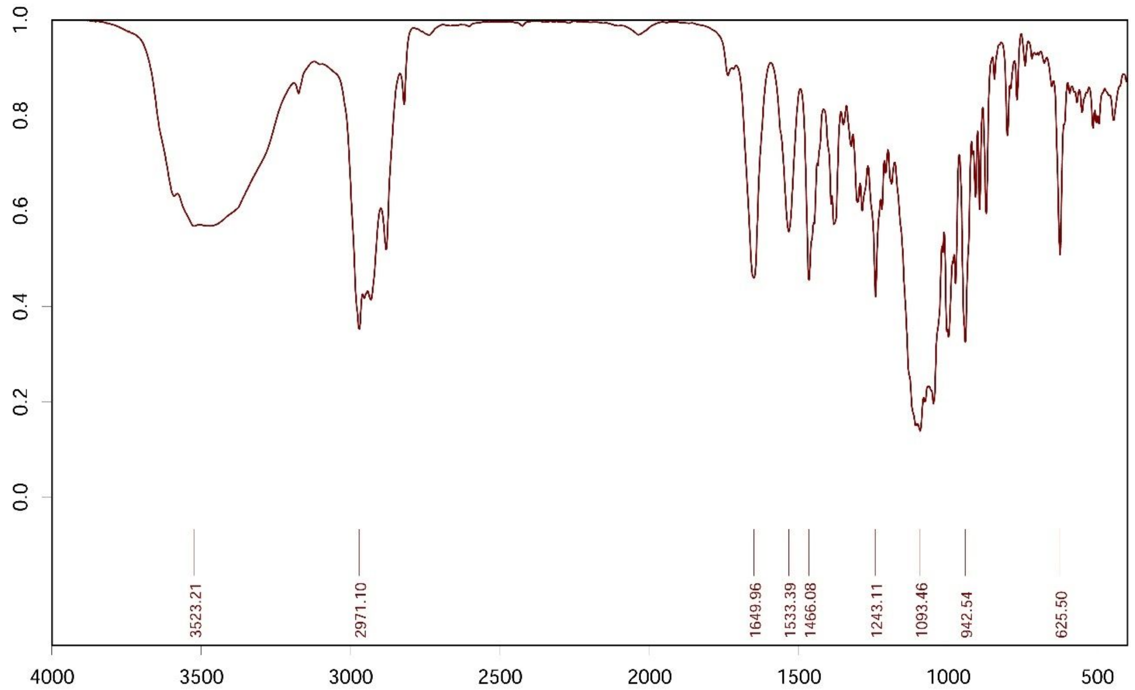

Figure S30. The FT-IR spectrum of **8** in the range of 4000-500 cm^{-1}.

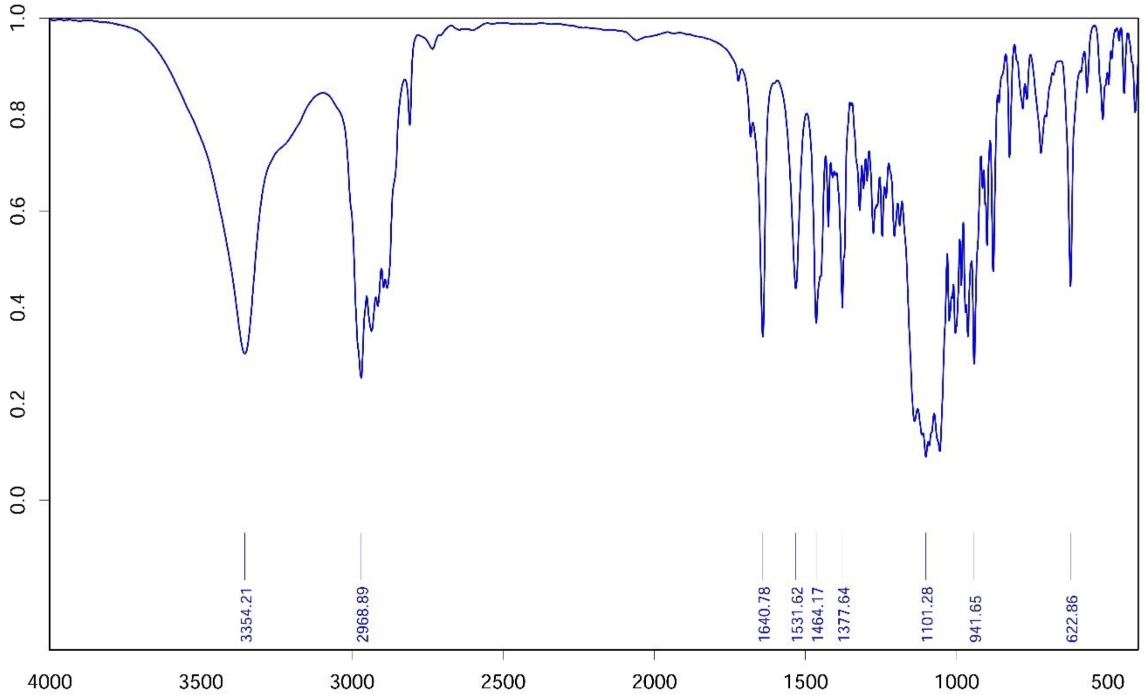

Figure S31. The FT-IR spectrum of **8** with the addition of $NaClO_4$ in the range of 4000-500 cm^{-1}.

X-ray measurements

The X-ray intensity data for compounds **2**, **5**, and **8** were collected using graphite monochromatic Mo K_{α} radiation on a four-circle κ geometry Xcalibur diffractometer with Sapphire2 area CCD detector. Data collections were made using the CrysAlisPro 1.171.42.93a. Integration, scaling of the reflections, corrections for Lorenz and polarization effects and absorption corrections were performed using the CrysAlisPro 1.171.42.93a program.[1] The structures were solved by the direct methods using SHELXT-2014/7[2] and refined using SHELXL-2018/3 program.[3] The hydrogen atoms joined to carbon atoms were introduced in their geometrical positions and treated as rigid. The H atoms involved in the hydrogen bonds were refined if they gave reasonable hydrogen bonds, otherwise they were constrained. The final difference Fourier maps showed no peaks of chemical significance. Details of the data collection parameters, crystallographic data and final agreement parameters are collected in Table S1. The hydrogen bonds are summarized in Table S2–S4. Visualizations of the structures were made with the Diamond 3.0.[4]

Table S1. Crystal data, collection data and structure refinement parameters for compounds **2**, **5**, and **8**.

		**2**	**5**	**8**
Formula		$C_{36}H_{61}N_3O_{10}\cdot H_2O$	$C_{39}H_{63}ClN_3NaO_{14}\cdot C_2H_3N$	$C_{36}H_{61}ClNNaO_{13}$
Molecular weight		713.89	897.41	774.29
Temperature (K)		100	100	100
Crystal system		Monoclinic	Monoclinic	Orthorhombic
Space group		$P2_1$	$P2_1$	$P2_12_12_1$
Unit cell dimensions	*a* (Å)	8.2504(2)	8.2439(4)	12.0123(4)
	b (Å)	17.1298(5)	22.8701(10)	14.9800(5)
	c (Å)	13.7271(3)	11.9581(6)	22.0196(6)
	β (°)	97.865(2)	90.789(5)	——
V (Å³)		1921.77(8)	2254.35(19)	3962.3(2)
Z		2	2	4
F(000)		776	960	1664
D_{cal} (g·cm^{-3})		1.234	1.322	1.298
θ range (°)		3.0–27.9°	3.0–26.9°	3.1–27.8°
μ (mm^{-1})		0.09 mm^{-1}	0.16 mm^{-1}	0.17 mm^{-1}
Crystal size (mm)		0.31 × 0.26 × 0.21	0.31 × 0.28 × 0.23	0.28 × 0.24 × 0.19
T_{min} / T_{max}		0.978/1.000	0.923/1.000	0.982/1.000
Total / unique / obs refls		42469/9508/7643	41081/10198/8912	20210/9341/6500
R_{int}		0.041	0.048	0.046

R [$F^2>2\sigma(F^2)$][a]	0.047	0.094	0.068
wR [F^2 all refls][a]	0.097	0.230	0.154
S	1.00	1.00	1.01
Flack parameter	−0.1 (2)	0.07(6)	0.04(5)
$\Delta\rho_{max}$, $\Delta\rho_{min}$ (eÅ^{-3})	0.28, −0.23	0.71, −0.47	0.76, −0.70

[a] $R=\Sigma \|\|F_o\|-\|F_c\|\|/\Sigma F_o$, $wR=\{\Sigma [w(F_o^2-F_c^2)^2]/\Sigma wF_o^4\}^{1/2}$; $w^{-1}=\sigma^2(F_o^2)+(aP)^2+bP$ where $P=(F_o^2+2F_c^2)/3$, a = 0.0391 and b = 0.5022 for **2**, a = 0.0803 and b = 11.2600 for **5** and a = 0.0503 and b = 3.7385 for **8.**

Table S2. Hydrogen bond geometry for **2** (Å, º).

D*—H···*A	***D*—H**	**H···*A***	***D*···*A***	***D*—H···*A***
O1—H1···O11	0.86 (3)	1.83 (4)	2.663 (3)	164 (3)
O3—H3*A*···O5	0.80 (4)	2.15 (4)	2.809 (3)	140 (3)
O3—H3*A*···O11	0.80 (4)	2.60 (4)	3.136 (3)	126 (3)
O9—H9*A*···O10	0.86 (4)	2.37 (4)	2.943 (3)	124 (3)
O9—H9*A*···O3	0.86 (4)	2.42 (4)	3.035 (3)	128 (3)
O11—H11*C*···O6	0.99 (4)	1.89 (4)	2.869 (3)	170 (3)
O11—H11*D*···O7	1.02 (4)	2.44 (3)	3.078 (3)	120 (2)
O11—H11*D*···O8	1.02 (4)	1.96 (4)	2.940 (3)	160 (3)

Table S3. Hydrogen bond geometry for **5** (Å, º).

D*—H···*A	***D*—H**	**H···*A***	***D*···*A***	***D*—H···*A***
O3—H3*A*···O9	0.88 (2)	1.95 (5)	2.737 (8)	149 (6)
O9—H9···O11^{i}	0.84 (3)	2.22 (8)	2.844 (8)	131 (9)
O9—H9···O13^{i}	0.84 (3)	2.53 (9)	3.094 (9)	126 (9)

Symmetry code: (i) *x*−1, *y*, *z*.

Table S4. Hydrogen bond geometry for **8** (Å, º).

D*—H···*A	***D*—H**	**H···*A***	***D*···*A***	***D*—H···*A***
N1—H1···O13a	0.86 (6)	2.60 (6)	3.303 (11)	140 (5)
N1—H1···O14a	0.86 (6)	2.08 (6)	2.859 (11)	151 (5)
N1—H1···O22b	0.86 (6)	2.32 (6)	3.152 (10)	163 (5)
N1—H1···O23b	0.86 (6)	2.35 (6)	3.018 (13)	135 (5)
O3—H3···O9	0.82 (6)	1.95 (6)	2.760 (5)	170 (6)

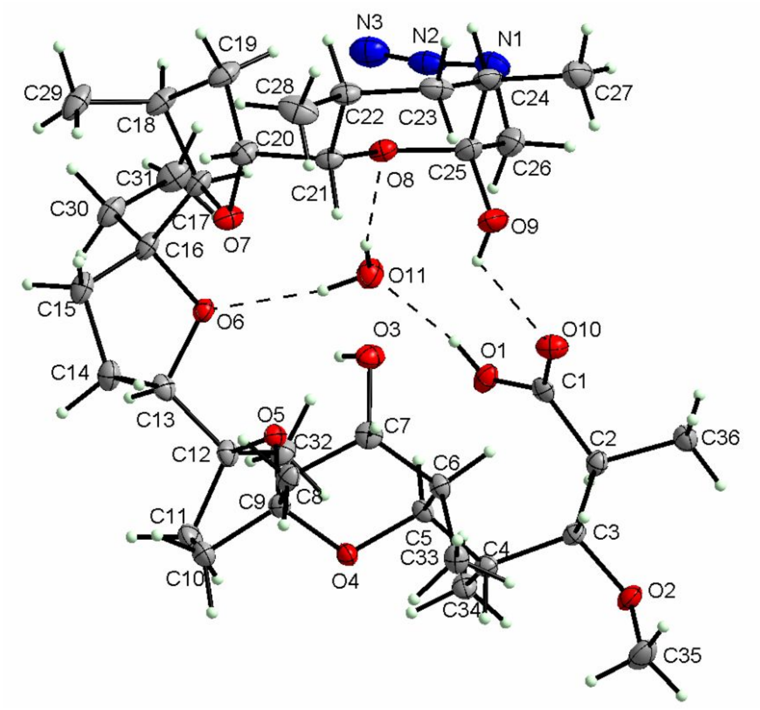

Figure S32. The crystal structure of the azide **2**. The anisotropic displacement parameters are presented at a probability level of 40%, and the H atoms are shown as circles with arbitrary radii.

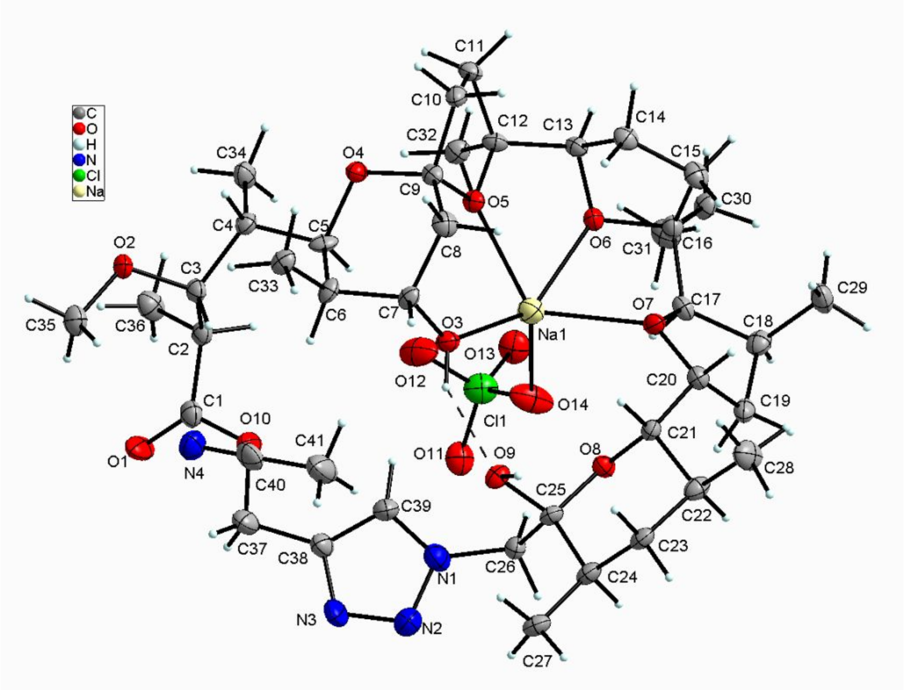

Figure S33. Crystal structure of the lactone **5**. The anisotropic displacement parameters are presented at a probability level of 40%, and the H atoms are shown as circles with arbitrary radii.

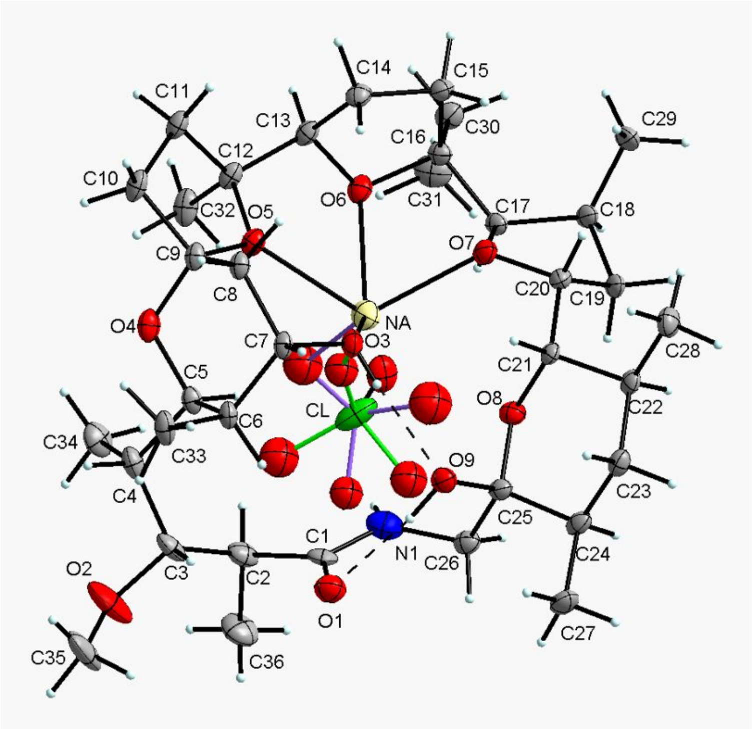

Figure S34. Crystal structure of the lactam **8**. In the structure, the perchlorate exist in two orientations, the superposition of which is shown in the picture. The anisotropic displacement parameters are presented at a probability level of 40% while the oxygen atoms of both orientations of perchlorate anion are shown at the 30% and the H atoms are shown as circles with arbitrary radii.

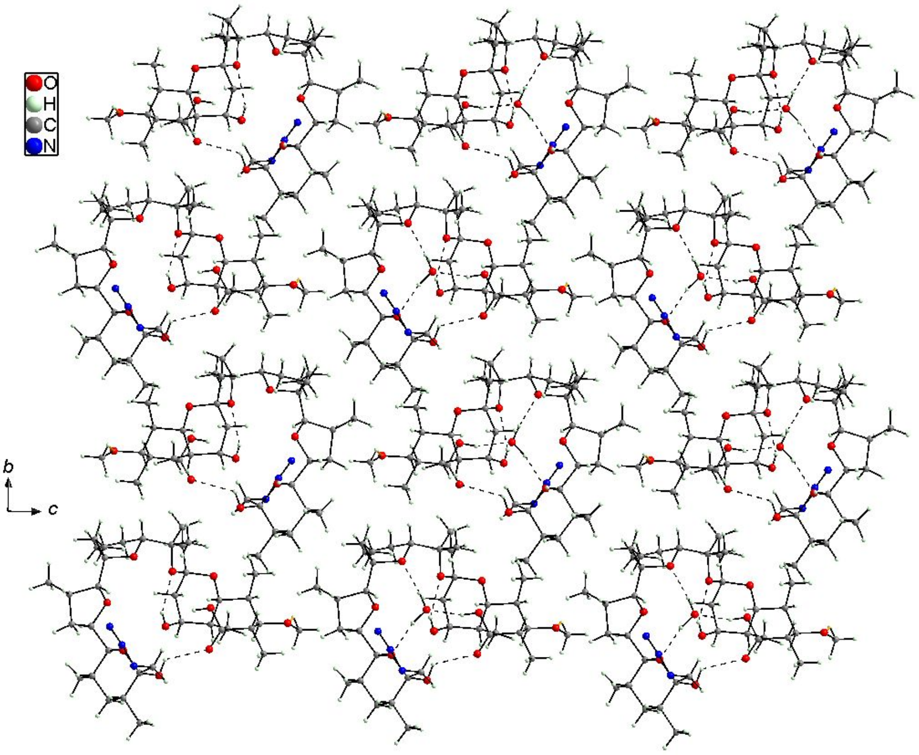

Figure S35. Packing along for **2**.

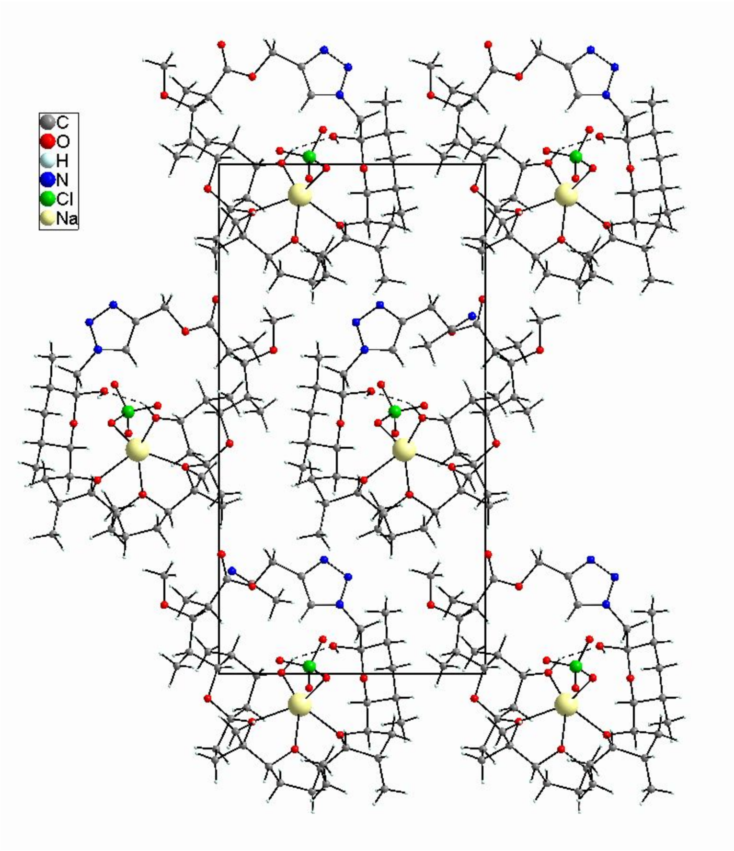

Figure S36. Packing along for **5**.

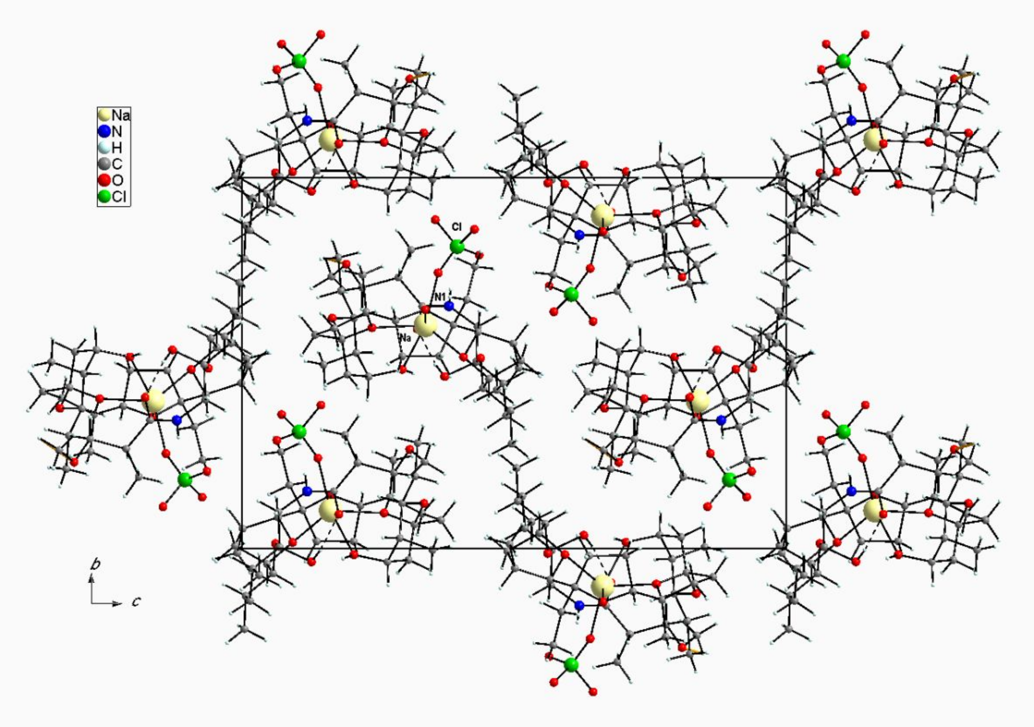

Figure S37. Packing along for **8**.

Literature

(1) Rigaku Oxford Diffraction. CrysAlis CCD and CrysAlis Red 1.171.38.43. 2015.

(2) Sheldrick, G. M. SHELXT – Integrated Space-Group and Crystal-Structure Determination. *Acta Crystallogr. A* **2015**, *71* (1), 3–8. https://doi.org/10.1107/S2053273314026370.

(3) Sheldrick, G. M. Crystal Structure Refinement with SHELXL. *Acta Crystallogr. C* **2015**, *71* (1), 3–8. https://doi.org/10.1107/S2053229614024218.

(4) K. Brandenburg, H. P. DIAMOND Version 3.0. Crystal Impact GbR, Bonn, Germany 2006.
