# Supplementary figures and images for: From Pseudocyclic to Macrocyclic Ionophores: Strategies toward the Synthesis of Cyclic Monensin Derivatives
Source: J Org Chem. 2025 Jan 10;90(3):1344–53. doi: 10.1021/acs.joc.4c02715 (PMC11773414; doi:10.1021/acs.joc.4c02715)

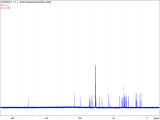

Supplement: Supplementary file 2 — jo4c02715_si_002.zip [file jo4c02715_si_002.zip › Spectra/Compound 2/13C NMR/pdata/1/thumb.png]

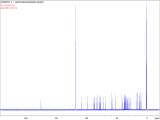

Supplement: Supplementary file 2 — jo4c02715_si_002.zip [file jo4c02715_si_002.zip › Spectra/Compound 3/13C NMR/pdata/1/thumb.png]

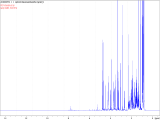

Supplement: Supplementary file 2 — jo4c02715_si_002.zip [file jo4c02715_si_002.zip › Spectra/Compound 3/1H NMR/pdata/1/thumb.png]

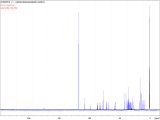

Supplement: Supplementary file 2 — jo4c02715_si_002.zip [file jo4c02715_si_002.zip › Spectra/Compound 4/13C NMR/pdata/1/thumb.png]

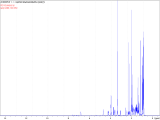

Supplement: Supplementary file 2 — jo4c02715_si_002.zip [file jo4c02715_si_002.zip › Spectra/Compound 4/1H NMR/pdata/1/thumb.png]

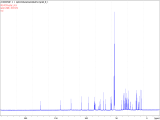

Supplement: Supplementary file 2 — jo4c02715_si_002.zip [file jo4c02715_si_002.zip › Spectra/Compound 5/13C NMR/pdata/1/thumb.png]

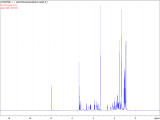

Supplement: Supplementary file 2 — jo4c02715_si_002.zip [file jo4c02715_si_002.zip › Spectra/Compound 5/1H NMR/pdata/1/thumb.png]
